# Phenotype-based screening rediscovered benzopyran-embedded microtubule inhibitors as anti-neuroinflammatory agents by modulating the tubulin–p65 interaction

**DOI:** 10.1038/s12276-022-00903-z

**Published:** 2022-12-12

**Authors:** Junhyeong Yim, Jaeseok Lee, Sihyeong Yi, Ja Young Koo, Sangmi Oh, Hankum Park, Seong Soon Kim, Myung Ae Bae, Jongmin Park, Seung Bum Park

**Affiliations:** 1 Department of Biophysics and Chemical Biology, Seoul National University, Seoul, 08826 Korea; 2 Department of Chemistry, Kangwon National University, Chuncheon, 24341 Korea; 3 CRI Center for Chemical Proteomics, Department of Chemistry, Seoul National University, Seoul, 08826 Korea; 4 Bio Platform Technology Research Center, Korea Research Institute of Chemical Technology, Daejeon, 34114 Korea; 5 Department of Medicinal Chemistry and Pharmacology, University of Science & Technology, Daejeon, 34114 Korea; 6 Kangwon Institute of Inclusive Technology, Kangwon National University, Chuncheon, 24341 Korea; 7 Present Address: Department of Dental Sciences, School of Dentistry and Dental Research Institute, Seoul National University, Seoul, 08826 Korea

**Keywords:** Drug discovery, Neurodegeneration

## Abstract

Neuroinflammation is one of the critical processes implicated in central nervous system (CNS) diseases. Therefore, alleviating neuroinflammation has been highlighted as a therapeutic strategy for treating CNS disorders. However, the complexity of neuroinflammatory processes and poor drug transport to the brain are considerable hurdles to the efficient control of neuroinflammation using small-molecule therapeutics. Thus, there is a significant demand for new chemical entities (NCEs) targeting neuroinflammation. Herein, we rediscovered benzopyran-embedded tubulin inhibitor **1** as an anti-neuroinflammatory agent via phenotype-based screening. A competitive photoaffinity labeling study revealed that compound **1** binds to tubulin at the colchicine-binding site. Structure–activity relationship analysis of **1**’s analogs identified SB26019 as a lead compound with enhanced anti-neuroinflammatory efficacy. Mechanistic studies revealed that upregulation of the tubulin monomer was critical for the anti-neuroinflammatory activity of SB26019. We serendipitously found that the tubulin monomer recruits p65, inhibiting its translocation from the cytosol to the nucleus and blocking NF-κB-mediated inflammatory pathways. Further in vivo validation using a neuroinflammation mouse model demonstrated that SB26019 suppressed microglial activation by downregulating lba-1 and proinflammatory cytokines. Intraperitoneal administration of SB26019 showed its therapeutic potential as an NCE for successful anti-neuroinflammatory regulation. Along with the recent growing demands on tubulin modulators for treating various inflammatory diseases, our results suggest that colchicine-binding site-specific modulation of tubulins can be a potential strategy for preventing neuroinflammation and treating CNS diseases.

## Neurodegeneration: A new approach to reduce brain inflammation

A screen for compounds that reduce inflammation in the brain has identified an effective compound with an unexpected mode of action, potentially opening a new therapeutic avenue for neurodegenerative diseases. Neuroinflammation contributes to the pathology of diseases like Alzheimer’s and Parkinson’s, but researchers have struggled to develop drugs targeting such inflammation. South Korean researchers led by Seung Bum Park of Seoul National University and Jongmin Park of Kangwon National University, Chuncheon, screened chemical compounds to identify those that mitigate the inflammatory response in cultured brain immune cells. They detected a compound that inhibits inflammation by disrupting the assembly of microtubules, multi-protein fibers that contribute to cellular infrastructure. Although this mechanism was unexpected, the researchers validated their findings in a mouse model of neuroinflammation, suggesting that this cellular pathway could offer a viable drug target.

## Introduction

Neuroinflammation in the central nervous system (CNS) has emerged as a critical contributing factor to neurodegenerative disorders such as Alzheimer’s disease (AD) and Parkinson’s disease (PD)^1–4^. The activation of microglia by diverse immunostimulatory processes (e.g., protein aggregation, pathogen infection, injury, or trauma) induces the secretion of neurotoxic cytokines and chemokines, resulting in neuronal dysfunction^5,6^, alteration in the neuronal environment^7,8^, disruption of the blood‒brain barrier, and eventually, severe impairment in cognitive functions^5,6,9^. Such debilitating consequences have created a significant demand for new chemical entities (NCEs) targeting neuroinflammation. More than 30 such drugs regulating inflammation have been investigated for treating neurodegenerative disorders (e.g., atomoxetine as noradrenaline reuptake inhibitors^10^; celecoxib as selective COX-2 inhibitors; CHF5074, flurizan, and ibuprofen as γ-secretase modulators^11–13^; neflamapimod, SB 239063, and PD 169316 as p38 MAPK inhibitors^14–17^; tetrandrine as an NF-κB inhibitor^18,19^; rosiglitazone, pioglitazone, and atorvastatin as PPARγ antagonists^20,21^). However, most failed to recover such patients’ cognitive functions^22^. The failures can be attributed to the complexity of neuroinflammatory processes and the lack of available knowledge.

Even though target-based drug discovery has been the leading approach for the past few decades due to its cost-effective throughput and well-validated process with target proteins, the pharmaceutical industry has suffered from a significant decrease in NCEs with new modes of action^23–25^. Therefore, phenotype-based drug discovery has drawn the attention of chemical biology researchers and the pharmaceutical industry. This approach focuses on discovering bioactive molecules that restore abnormal disease-relevant phenotypes without knowing their exact modes of action^24,26^. Using the phenotype-based drug screening process, we might reveal undruggable protein targets, unknown protein modulators, or proteins having novel modes of action to treat various diseases with high unmet medical needs. Therefore, phenotype-based screening could facilitate the unbiased discovery of effective NCEs for regulating neuroinflammation.

Herein, we present a benzopyran-embedded small molecule discovered via phenotype-based screening as a potent neuroinflammatory inhibitor. Interestingly, we rediscovered our previously reported tubulin-modulating compound **1** as an initial hit compound with anti-inflammatory activity in murine microglial BV-2 cells^27^. Through a structure–activity relationship (SAR) study, we generated SB26019 with improved efficacy in managing neuroinflammation. Mechanistic studies revealed the crucial role of tubulin monomers as regulators of p65 via SB26019-mediated modulation of protein‒protein interactions in cellular neuroinflammatory models. These observations suggest a new role of tubulin in anti-inflammation beyond its traditional functions. Mouse model studies further validated the role of SB26019 in suppressing microglial activation. The in vivo efficacy of SB26019 via intraperitoneal (IP) injection confirmed its therapeutic potential as an NCE for neuroinflammatory regulation. It is worth mentioning that colchicine, a tubulin modulator, has been presented as a therapeutic agent in recent clinical trials for various diseases, including Behçet’s disease^28^, atrial fibrillation after cardiac surgery^29^, COVID-19 infection^30–32^, cardiovascular disease^33–36^, inflammatory dermatitis^37^, and hepatocyte-mediated myeloid cell inactivation^38^. In the context of this emerging attention on tubulin modulators, our findings could expand the therapeutic potential of tubulin modulation for treating neurodegenerative diseases.

## Materials and methods

### Griess assay

Cellular secretion of nitric oxide (NO) was quantified with the Griess assay. BV-2 cells were seeded on a clear flat bottom TC-treated 96-well plate (Corning; #3598) and sealed with breathable sealing tape (Axygen; BF-400-S). After 18 h, BV-2 cells were treated with compounds in the absence or presence of 100 ng/mL LPS (Sigma; L4391). After 24 h, 50 μL of the cell culture media was mixed with the same volume of Griess reagent {0.1% naphthylethylenediamine dihydrochloride (Sigma; #222488) and 1% sulfanilamide (Sigma; S9251) in 4% phosphoric acid (Daejung; #6532-4405)}. Absorbance at 550 nm was measured using a microplate reader (BioTek; Synergy). Sodium nitrite (Alfa Aesar; A18668) was used for standard curve generation.

### Cell viability assay

A total of 40,000 cells were seeded on a 96-well plate and incubated for 18 h. After 6-, 12- and 24-h compound treatment, 10 μL of EZ-Cytox solution (Dogen; EZ-BULK150) was added to the 96-well plate. After 30 min, the absorbance at 455 nm was measured using a microplate reader (BioTek; Synergy).

### qPCR

After cell lysis with RLT buffer (Qiagen), RNA was extracted using the RNAeasy PLUS Mini Kit (Qiagen; #74136). The resulting cDNA was generated using the AccuPower CycleScript RT PreMix (dT20) kit (Bioneer; K2044) on a C1000 Touch Thermal Cycler (Bio-Rad; #1841000). qPCR was performed using a KAPA SYBR® FAST qPCR Master Mix (2×) Universal Kit (Kapa; KK4605) on a StepOne Real-Time PCR system (Applied Biosystems; #4376357). The cycling threshold value of the endogenous control gene GAPDH was subtracted from the cycling threshold value of each target gene to calculate the shift in cycling threshold (ΔCT). The relative expression of each target gene is denoted as the “fold change” comparable to that of vehicle-treated samples (2−ΔCT). The primer sequence information for this analysis is shown in Supplementary Table 3.

### TNF-α ELISA

BV-2 and RAW264.7 cells were treated with compounds in the absence or presence of LPS (100 ng/ml). After 24 h, TNF-α levels in the culture medium were measured using a TNF-α ELISA kit (R&D Systems; DY410) according to the manufacturer’s protocol. Briefly, capture antibodies were coated on 96-well Maxisorp Nunc-Immuno plates (Thermo; #439454) for 1 h and washed with PBS containing 0.05% Tween-20 (PBST) three times. The plates were blocked with 1% BSA-PBS for 1 h and washed with PBST three times. Culture medium was added to the 96-well plate for 1 h and washed with PBST three times. Biotinylated detection antibody was added to the 96-well plate for 1 h and washed with PBST three times. Streptavidin–horseradish peroxidase (HRP) was added to the plate for 20 min and washed with PBST three times. After 20 min of incubation with TMB substrate (Thermo; #002023), TMB oxidation was stopped by adding 2 N H_2_SO_4_. The absorbance at 450 nm, 540 nm, and 570 nm was measured using a microplate reader (BioTek; Synergy). The following formula was used to calculate the net signal: OD_450nm_–(OD_540nm_ + OD_570nm_)/2.

### Immunofluorescence staining

To monitor p65 translocation, J774A.1 cells were treated with compounds for 1 h, followed by LPS (100 ng/ml) treatment for 30 min. The cells were fixed with 4% paraformaldehyde (Sigma; #158127) in PBS (w/v) for 30 min at room temperature. After washing with 1× PBS (Welgene; ML008-02) three times, the fixed cells were permeabilized with PBS containing 0.1% Triton X-100 for 10 min. The cells were washed with 1× PBS three times and then blocked with 4% bovine serum albumin (MP Biomedicals; #0216006980) in PBS (w/v) for 1 h. The cells were incubated with mouse anti-p65 antibody (Santa Cruz; sc-8008, 1:200 diluted in 1% BSA–PBS) at 4 °C overnight and washed with PBS three times. FITC-labeled goat anti-mouse IgG antibody (Abcam; ab6785, 1:200 diluted in 1% BSA–PBS) was added to the sample and incubated for 1 h at room temperature. The remaining antibodies were finally washed three times with PBS. Nuclei were stained with Hoechst 33342 (Thermo; #62249). The cellular p65 proteins were imaged by fluorescence microscopy (GE Healthcare; Delta Vision) and quantified by the line profiling method.

### Photoaffinity-based competition assay

Porcine tubulin (Cytoskeleton; 10 μM; T238P-A) was incubated in the absence or presence of colchicine (TCI; C0380), vinblastine (Sigma; V1377), and Taxol (TCI; P1632). A photoaffinity-based target ID probe of **1** (5 μM) was added and incubated for 75 min at room temperature. UV light (365 nm) was irradiated for 5 min to generate a covalent bond between the probe and tubulins. A click reaction between the acetylene group of target ID probe **1** and Cy5-azide was conducted at room temperature for 1.5 h. For the click reaction, 5% *t*-BuOH (TCI; B0706), 1 mM CuSO_4_ (Sigma; #209198), 100 μM TBTA {tris[(1-benzyl-1H-1,2,3-triazol-4-yl)methyl]amine, Sigma; #678937}, 2 mM TCEP {tris(2-carboxyethyl) phosphine hydrochloride, Alfa; J60316}, and 40 μM Cy5-azide (Lumiprobe; #33030) were added. Tubulin protein was analyzed by SDS‒PAGE, and Cy5-labeled tubulin was measured by a fluorescent gel scanner (Azure Biosystems; Sapphire). The whole loading level of tubulins was visualized by silver staining and imaged by ChemiDoc (Bio-Rad).

### Molecular modeling

Molecular structure optimization was performed with MM2 minimization using ChemBio3D Ultra v11.0.1. and DFT calculation (6–31 G basis set) using Gaussian 09 W. Computational binding analysis between lead compounds and tubulin was performed with Discovery Studio Client v16.1.0.15350 using the cocrystal structure of colchicine and tubulin (PDB code: 1SA0). The binding site of tubulin was defined from the PDB database. Docking simulation was performed with the C-DOCKER module. To compare the binding energy between tubulin and each compound, absolute values of C-DOCKER interaction energy were used (Table 1). A 2-D interaction module was used to find the interactions between compound **1** and amino acid residues of the tubulin complex (1SA0 full structure). Docked structures were visualized using UCSF Chimera v1.14.

**Table 1:** Structure–activity relationship (SAR) study by modifications at the R_1_ and R_2_ positions of compound **1**.


Cpd.	R_1_	R_2_	IC_50_ of Griess Assay	% NO inh. at 10 μM	% Viab. at 10 μM	- C-Docker Interaction energy
**1**	Methyl	4-Chloro-2-methylphenyl	5.22	69.4	102.4	35.91
**2**	Methyl	4-Fluoro-2-methylphenyl	5.94	65.3	105.3	36.24
**3**	Methyl	4-Fluorophenyl	22.28	9.9	88.0	32.96
**4**	Methyl	2-Methylphenyl	7.19	73.3	106.1	36.85
**5**	Methyl	2,5-Dimethylphenyl	9.89	46.9	99.7	38.37
**6**	Methyl	2-Methoxyphenyl	5.09	87.1	113.8	38.15
**7**	Methyl	Quinolin-8-yl	5.01	98.0	101.5	39.23
**8** (SB26019)	Methyl	Dibenzofuran-4-yl	1.13	101.7	108.0	40.73
**9**	Methyl	Dibenzothiophen-4-yl	1.00	90.0	103.6	40.82
**10**	Methyl	Thianthren-1-yl	13.12	36.8	102.1	37.07
**11**	Methyl	1,1-Biphenyl-3-yl	6.71	61.6	106.1	42.74
**12**	Ethyl propionate	4-Chloro-2-methylphenyl	7.45	72.3	105.6	48.03
**13**	Ethyl propionate	Dibenzofuran-4-yl	1.79	101.2	111.4	46.58
Nocodazole			13.44	54.9	93.8	
Colchicine			4.20	51.8	113.8	

### Western blot analysis

The proteome was analyzed by SDS‒PAGE and transferred to PVDF membranes (Bio-Rad; BR162-0177). The membranes were blocked with 2% BSA in Tris-buffered saline containing 0.1% Tween-20 (TBST) for 1 h. To detect the desired proteins, the membranes were incubated overnight at 4 °C with the primary antibodies—1:1000 dilution of α-tubulin (CST; #3873), β-tubulin (CST; #2146), IκB (CST; #4814), caspase-1 (CST; #24232), cleaved caspase-1 (CST; #89332), IL-1β (CST; #31202), cleaved IL-1β (CST; #63124), acetylated α-tubulin (CST; #5335), p65 (Abcam; ab16502), FLAG (Abcam; ab49763); and 1:2,000 dilution of GAPDH (CST; #2118), β-actin (CST; #4970), and LMNB1 (Abcam; ab16048). After washing with TBST, the resulting membranes were exposed to HRP-conjugated secondary antibody—1:5000 dilution of anti-rabbit (CST; #7074) and anti-mouse (CST; #7076)—for 1 h at room temperature. After washing with TBST, the membranes were incubated with an enhanced chemiluminescence (ECL) prime kit (Cytiva; RPN2232). Chemiluminescent signals from the desired proteins were detected using ChemiDoc.

### Subcellular fractionation

After SB26019 treatment for 6 h, J774A.1 cells underwent subcellular fractionation with NE-PER nuclear and cytoplasmic extraction reagents (Thermo; #78833) according to the manufacturer’s protocol. The resulting samples were analyzed by SDS‒PAGE and western blotting. The purity of the nuclear and cytosolic fractions was confirmed by the cytosolic marker protein GAPDH and the nuclear marker protein LMNB1, respectively.

### Immunoprecipitation analysis

After a 1-h incubation in the absence or presence of SB26019, cells were harvested and lysed for 1 h on ice with IP lysis buffer {50 mM Tris–HCl pH 8.0 (Acros; #77-86-1), 200 mM NaCl, 0.5% NP40 (Sigma; #I8896), and 1× protease inhibitor cocktail (Roche; #11873580001)}. The protein concentration was measured using a bicinchoninic acid (BCA) protein assay kit (Thermo; #23225). Then, 150 μg of the lysates were incubated with 1.5 μg of anti-p65 antibody (Abcam; ab16502), anti-IκB antibody (CST; #4814), or α-tubulin antibody (CST; #3873) at 4 °C overnight. Subsequently, the proteins of interest were precipitated using protein A and G beads (Santa Cruz; sc-2001, sc-2002). Samples were analyzed with western blotting using anti-p65, IκB, and α-tubulin primary antibodies.

### Tubulin monomer/polymer isolation

After transfection with pCMV6 mock vector (Origene; PS100001) or α-tubulin plasmid for 1 day, RAW264.7 cells were treated with colchicine or SB26019 for 6 h. To separate soluble tubulin monomers from insoluble tubulin polymers, cells were washed very gently with PBS and incubated for 10 min at room temperature with a microtubule-stabilizing buffer {10% Tris–HCl (pH 6.8) (Acros; #77-86-1), 10% glycerol (Biosesang; GR1018-500-00), 1 mM EGTA (Sigma; E3889), 1 mM MgSO_4_ (Sigma; M2773), 0.1 mM EDTA (Acros; #409971000), and 1× protease inhibitor cocktail} containing additional 0.1% Triton X-100 (Sigma; T8787). The remaining insoluble tubulin polymers on the culture dish were scraped with microtubule-stabilizing buffer containing an additional 0.5% SDS (Sigma; L3771). Tubulin monomer/polymer isolation was confirmed by western blot with anti-α-tubulin and anti-FLAG primary antibodies.

### Tubulin polymerization assay

The tubulin polymerization inhibition efficacy of the compounds was measured by a tubulin polymerization assay kit (Cytoskeleton; BK006P) according to the manufacturer’s manual. Absorbance at 340 nm was measured to observe the formation of tubulin polymers using a microplate reader (Biotek; Synergy).

### Animal experiments

Eight-week-old C57BL/6 female mice (20–25 g) from CJR (Charles River, Japan) were supplied by Orient Bio, Inc. The animals were maintained in temperature- and humidity-controlled conditions with a 12-h light and 12-h dark cycle. All animal experiments were approved by the IACUC (Institutional Animal Care and Use Committee) of Seoul National University with the approval number “SNU-200122-5-1”. In vivo experiments were carried out under the guidelines in the US National Institutes of Health Guide for the Care and Use of Laboratory Animals.

### LPS neuroinflammation model

All experiments were carried out with 10-week-old female C57BL/6 mice (20–25 g). The animals were divided into six experimental groups of three mice in each experiment: Group 1, treated with vehicle; Group 2, treated with SB26019 {2 mg/kg body weight (mpk)}; Group 3, treated with SB26019 (5 mpk); Group 4, treated with LPS and vehicle; Group 5, treated with LPS and SB26019 (2 mpk); and Group 6, treated with LPS and SB26019 (5 mpk). SB26019 (2 or 5 mpk) or vehicle {distilled water containing 5% DMSO and 40% polyethylene glycol 400 (TCI; N0433)} was administered with IP injection daily for 4 days. Five mpk of LPS (Sigma; L2880) in PBS was administered by IP injection on Day 2 for a single challenge. All mice were sacrificed on Day 5.

### Histological analysis

Mice were anesthetized with a mixture of alfaxalone and xylazine (5:1 ratio) and transcardially perfused with cold PBS. Mouse brains were harvested and fixed using 10% NBF (Sigma; HT501128) for 2 h. After washing with PBS for 30 min three times, fixed brains were embedded in OCT compound (Leica; #3801480) for frozen coronal sectioning at a thickness of 7 μm. To detect microglial activation, brain sections were blocked with 5% donkey serum in PBS (Jackson Laboratory; #017-000-121) for 1 h and then incubated with goat anti-Iba-1 antibody (Abcam; ab5076, 1:100 dilution) and rabbit anti-TMEM119 antibody (Abcam; ab209064, 1:100 dilution) at 4 °C overnight. The sections were visualized with Alexa Fluor 488-conjugated donkey anti-goat IgG antibody (Jackson Laboratory; #705-545-003, 1:200 dilution) and Alexa Fluor 568-conjugated donkey anti-rabbit IgG (Abcam; ab175693, 1:300 dilution). Confocal microscopy (Zeiss; LSM800) in the Korea Mouse Phenotyping Center (KMPC) was used to analyze the Iba-1-positive area of sections.

### Microglia isolation

The perfused brains were minced with a 10 T scalpel blade. The minced brain was incubated with dissociation medium {DMEM/F12 (Gibco; #12634-010) containing 1 mg/ml papain (Sigma; P4762), 1.2 U/ml dispase II (Sigma; D4693), 20 U/ml DNase I (Sigma; D5025)} for 20 min, followed by the addition of neutralization medium {DMEM/F12 containing 1× penicillin‒streptomycin (Gibco; #15140122), 4.5 μg/ml glucose (Sigma; A11156), and 10% FBS (Gibco; #16000-044)}. Softened brains were separated into cells by serial pipetting with 5.0, 1.0, and 0.2 ml tips. The separated cells were strained with a 40 µM cell strainer (Falcon; #352340) and centrifuged with 30%, 37%, and 70% Percoll solution (GE healthcare; # 17-0891-01). Microglia in the interphase between 70% and 37% Percoll solution was transferred and washed with HBSS media. mRNA was extracted from the microglia and analyzed with qPCR.

### Statistics

Statistical analyses of in vitro experiments and LPS-induced in vivo experiments were performed with Student’s *t*-test using GraphPad Prism. Data are represented as the mean ± SD (standard deviation), as indicated in the individual figure legends. The *P* value is also indicated in the individual figure legends.

## Results

### Phenotype-based screening for an anti-neuroinflammatory agent and identification of its binding site

**Fig. 1:**
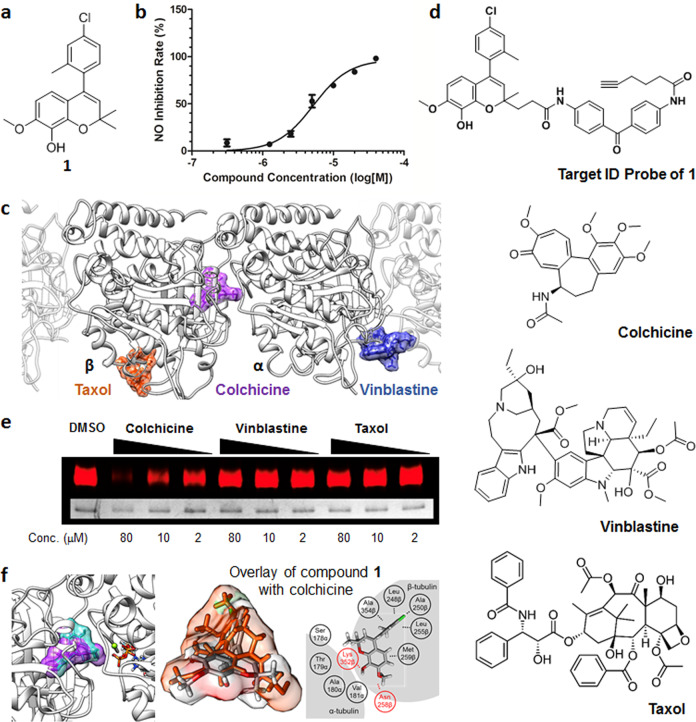
Neuroinflammatory regulation and binding site of tubulin inhibitor. **a** Chemical structure of initial hit compound **1**. **b** Dose-dependent inhibition of cellular NO release by **1** against lipopolysaccharide (LPS) treatment in BV-2 microglial cells (*n* = 6). **c** Binding sites of known tubulin modulators; Taxol (orange), colchicine (purple), and vinblastine (blue). **d** Chemical structures of the target ID probe of **1** and known microtubule modulators. **e** Photoaffinity-based competition assay showed that **1** bound to the colchicine-binding site. Purified tubulin was cross-linked with the target ID probe of **1** under UV irradiation in the presence or absence of known tubulin modulators. Tubulin was further labeled with Cy5-azide through a Click reaction. **f** Docking analysis of compound **1** using cocrystal structures of tubulin with its binders. Left panel: colchicine (purple) and **1** (cyan) bind to the same site of tubulin. Middle panel: Structural similarity of compound **1** (gray) and colchicine (orange). Right panel: Expected interactions between **1** and amino acid residues of tubulin.

Microglia are tissue-resident macrophages involved in CNS immunosurveillance^3,39^. Microglial activation induces proinflammatory cytokine tumor necrosis factor α (TNF-α) expression, resulting in neural toxicity^5,6^. Therefore, the inhibition of microglial activation is critical for regulating neuroinflammation. To discover anti-neuroinflammatory NCEs, we monitored the cellular release of nitric oxide (NO) in response to lipopolysaccharide (LPS) treatment as a mimic of pathogenic activation of innate immune systems upon compound treatment in BV-2 cells using the Griess assay. From this phenotype-based screening of a privileged substructure-based diversity-oriented synthesis (pDOS) library^40,41^ containing 6000 drug-like small molecules, we identified benzopyranyl polyheterocycle **1** as one of the initial hits with moderate anti-neuroinflammatory activity (Fig. 1a and Supplementary Fig. 1). Hit compound **1** inhibited LPS-mediated NO release in BV-2 microglial cells down to 70% at a 10 μM concentration, with a half-maximal inhibitory concentration (IC_50_) of 5.22 μM (Fig. 1b).

Intriguingly, compound **1** was previously reported as a tubulin polymerization inhibitor^27^. Considering that colchicine has been used to treat gout by inhibiting inflammation^42–45^, we hypothesized that initial hit compound **1** might regulate neuroinflammation by controlling tubulin dynamics. To improve the anti-inflammatory efficacy of **1**, we first identified the exact binding site of **1** on tubulin. In fact, tubulin modulators with different binding sites (Fig. 1c and Supplementary Fig. 2) are known to induce different thermal stability shifts to the tubulin protein due to thermal proximity coaggregation^46,47^. A cellular thermal shift assay (CETSA) confirmed that tubulin modulators, including colchicine, vinblastine, and paclitaxel, have different heat resistances, as reported previously (Supplementary Fig. 3a). However, compound **1** showed slight thermal sensitization in CETSA, unlike any of the three representative tubulin binders. We then investigated the binding site of compound **1** using a photoaffinity-based target ID probe. In the presence or absence of tubulin binders (colchicine, vinblastine, and paclitaxol), the target ID probe of **1** (Fig. 1d) was incubated with recombinant tubulin protein. The subsequent UV irradiation for photocrosslinking tubulin with the target ID probe of **1** and the bioorthogonal labeling of Cy5-azide with the Click reaction visualized the competitive fluorescence labeling patterns of each tubulin modulator. Out of three tubulin modulators, colchicine outcompeted the target ID probe of **1** in a dose-dependent manner, suggesting that compound **1** and colchicine share the same binding site (Fig. 1e). We also observed a similar competition pattern of the target ID probe of **1** with combretastatin—a tubulin binder sharing the colchicine-binding site (Supplementary Fig. 3b). Molecular docking analysis provided further confirmation of the above and showed a perfect overlay of the binding mode of **1** with colchicine (Fig. 1f).

### A structure–activity relationship study generates SB26019 as a potent anti-neuroinflammatory agent

To enhance the inhibitory potency of initial hit **1**, a series of its analogs were synthesized and evaluated. Initial screening indicated that the derivatization at the R_a_, R_b_, and R_c_ positions (**1a**–**1c**) resulted in a loss of anti-inflammatory efficacy (Supplementary Table 1). Therefore, we modified the R_1_ and R_2_ positions of compound **1** and constructed a 12-membered focused library to yield more potent neuroinflammatory inhibitors. Using a tubulin polymerization assay, we monitored the rate of tubulin polymerization in the presence and absence of compounds by measuring *V*_max_; nocodazole (the microtubule inhibitor) and our analogs showed a reduced *V*_max_ of microtubule polymerization compared to the dimethylsulfoxide (DMSO) control (Supplementary Table 2 and Supplementary Fig. 4). Subsequent evaluation of the anti-neuroinflammatory activities of these analogs confirmed that the 2,4-disubstituted aryl ring on the R_2_ position is critical for anti-neuroinflammatory activity (**1**–**6**), as indicated in Table 1 and Supplementary Fig. 5. The anti-neuroinflammatory activity was enhanced upon replacing phenyl rings with fused heteroaromatic rings (**7**–**9**), except for a thianthren-1-yl-substituted molecule (**10**). Conversion of the methyl group at the R_1_ position to ethyl propionate (**12** and **13**) did not affect the efficacy compared to the original compounds **1** and **8**, respectively. Tubulin inhibition and anti-neuroinflammatory assays indicated that 7-methoxy-8-hydroxybenzopyran substituted with a 4-dibenzofuran-4-yl moiety (**8**) significantly inhibited tubulin polymerization and cellular NO release. Furthermore, compound **8** showed one of the lowest IC_50_ values, as indicated by the Griess assay, without considerable cytotoxicity (Supplementary Fig. 6). Therefore, we selected compound **8** as the lead compound and named it SB26019 for further biological studies.

To provide a rationale for the correlation between anti-neuroinflammatory and microtubule inhibition activities, in silico docking of the focused library compounds (**1**–**13**, Table 1) with the colchicine-binding domain of tubulin was performed using the C-DOCKER module in Discovery Studio and represented by the absolute value of the C-DOCKER interaction energy. The tubulin–small molecule interaction energy and the efficacy of compounds in inhibiting cellular NO release were positively correlated (Pearson correlation coefficient, *r* = 0.545), while the IC_50_ value of the Griess assay negatively correlated with the absolute value of tubulin–small molecule interaction energy (*r* = ‒0.530) (Supplementary Fig. 7). These docking results guided our subsequent experiments to focus on our hypothesis that tubulin dynamics regulate neuroinflammation. To further validate the correlation between the anti-neuroinflammatory effect and tubulin polymerization inhibition, we conducted a compensated Griess assay of SB26019 in the absence or presence of the tubulin stabilizer Taxol; the anti-neuroinflammatory activity of SB26019 was attenuated in the presence of Taxol in a dose-dependent manner, indicating that tubulin polymerization inhibition triggered the anti-neuroinflammatory effect of SB26019 (Supplementary Fig. 8 and Supplementary Fig. 9).

### SB26019 regulates NF-κB activation by inducing monomeric α-tubulin formation

**Fig. 2:**
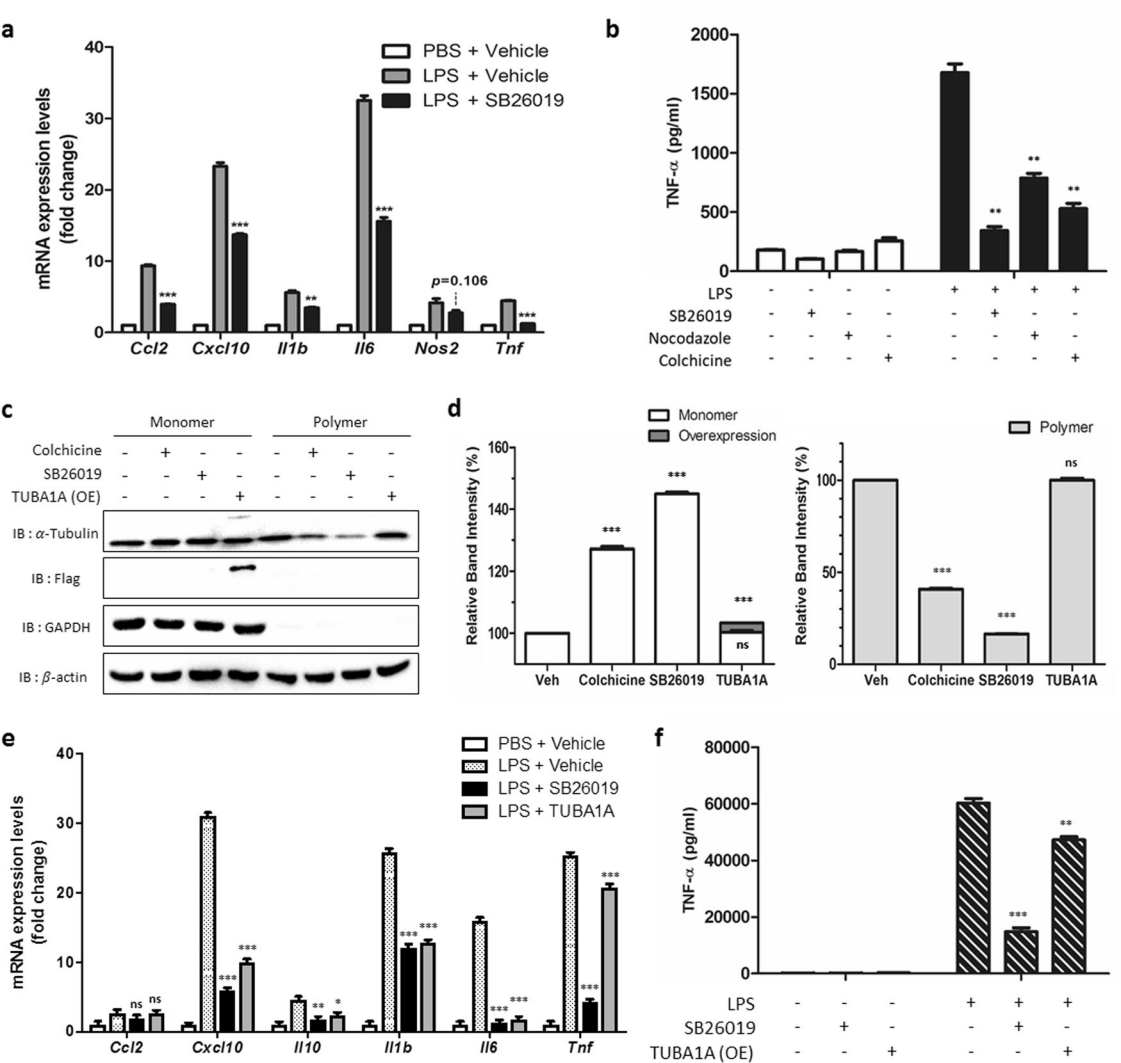
SB26019 has anti-inflammatory activity via the accumulation of tubulin monomers. SB26019 (10 μM for 6 h) regulated the mRNA levels (**a**) and protein levels (**b**) of downstream genes of NF-κB in BV-2 murine microglial cells more efficiently than the other known tubulin modulators (*n* = 3). **c** Both tubulin modulators (10 μM for 6 h) and transient Flag-tagged α-tubulin overexpression increased the total amount of tubulin monomers. **d** Quantification of the amount of tubulin polymer vs. monomer in (**c**). Tubulin modulators (10 μM for 6 h) and transient Flag-tagged α-tubulin overexpression proportionally regulated the NF-κB signaling pathway at the mRNA (**e**) and protein levels **f** in RAW264.7 murine macrophage cells (*n* = 3). Data are presented as the mean ± SD (ns, not significant, *p* > 0.05; *p < 0.05; **p < 0.01; ***p < 0.001).

NF-κB is a central mediator that induces proinflammatory cytokines, including TNF-α, IL-1β, IL-6, and chemokines^48^. Since colchicine and other molecules sharing the colchicine-binding site have been reported to act as NF-κB modulators^49,50^, we monitored the LPS-induced NF-κB signaling pathway upon SB26019 treatment in BV-2 microglial cells; the NF-κB-mediated regulation of proinflammatory cytokines and chemokines was analyzed by quantitative polymerase chain reaction (qPCR). SB26019 suppressed the production of inflammatory marker genes, such as Ccl2, Cxcl10, Il-1β, Il-6, Nos2, and Tnf, in a dose-dependent manner (Fig. 2a and Supplementary Fig. 10). The expression level of the proinflammatory cytokine TNF-α was also monitored with SB26019 treatment. LPS treatment induced the secretion of TNF-α into culture media, whereas SB26019 and colchicine-domain binders (nocodazole and colchicine itself) reduced TNF-α levels to <50% compared to that of the DMSO control (Fig. 2b).

Based on the SAR study and NF-κB pathway analysis, we hypothesized that upregulated tubulin monomers by SB26019 may regulate the NF-κB signaling pathway. To examine whether SB26019 treatment induces the transition between tubulin monomers and their polymers, we extracted tubulin monomers and polymers from the cells and analyzed them by western blotting. Considering the previous report that α-tubulin monomers mediate the regulation of the inflammatory transcription factor activator protein 1 (AP-1)^51^, we focused on α-tubulin subunits rather than β subunits. Treatment with tubulin inhibitors (colchicine and SB26019) increased the total α-tubulin monomers in RAW264.7 murine macrophage cells (Fig. 2c). Notably, treatment with the more potent anti-inflammatory agent SB26019 (Table 1, IC_50_ of 1.13 μM) produced more significant amounts of α-tubulin monomers and fewer tubulin polymers than the less potent anti-inflammatory regulator colchicine (Table 1, IC_50_ of 4.20 μM) (Fig. 2d). This observation also suggested a positive correlation between anti-inflammatory activity and the number of tubulin monomers in cells. Subsequent mRNA analysis and ELISA further confirmed that α-tubulin monomers regulate NF-κB activation. The expression levels of LPS-induced inflammatory cytokines and chemokines were downregulated as α-tubulin monomers were increased in cells upon SB26019 treatment or α-tubulin overexpression (Fig. 2e, Supplementary Figs. 11 and 12). Interestingly, we observed no cell cycle arrest by SB26019 in this experimental condition, demonstrating the increased cellular α-tubulin level by SB26019, not the cytotoxicity of SB26019, attributed to its anti-inflammatory activity (Supplementary Fig. 13). The TNF-α expression level was also regulated by the increased level of α-tubulin monomers (Fig. 2f). Even though FLAG-tagged α-tubulin transfection increased only 3% of the total α-tubulin monomers, its anti-inflammatory effect was comparable to SB26019 treatment (Fig. 2d, e).

### SB26019-induced α-tubulin monomer inhibits p65 translocation

**Fig. 3:**
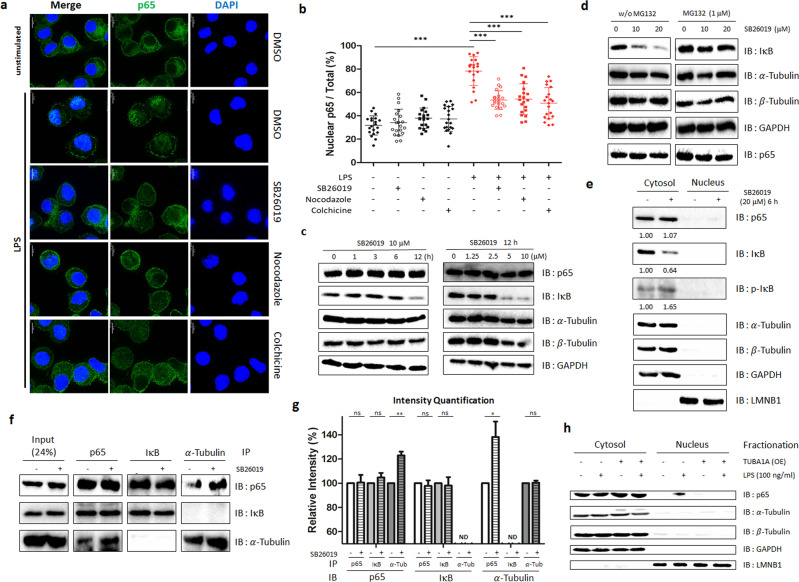
Tubulin monomers induced by tubulin modulators inhibit p65 nuclear translocation to exert anti-inflammatory effects. **a** Treatment with tubulin modulators (10 μM for 1 h) inhibited p65 nuclear translocation in J774A.1 cells. p65 and the nucleus were stained with a fluorescently labeled antibody and Hoechst 33342, respectively. **b** Quantification of fluorescence images (**a**) (*n* = 20). **c** Dose- and time-dependent IκB degradation by SB26019 without affecting the total amount of p65 in J774A.1 cells. **d** SB26019 (6 h) induced the degradation of IκB, which was ameliorated by treatment with MG132, a proteasome inhibitor. **e** Subcellular localization of p65 after SB26019 treatment in J774A.1 cells. p65 remained in the cytosol after its dissociation from IκB. **f** Tubulin-p65 interaction increased after 1 h of treatment with SB26019 (10 μM). Increased levels of tubulin bound to p65, generating the tubulin–p65 complex. The tubulin–p65 complex is hypothesized to be the reason for the inhibition of p65 nuclear translocation. **g** Quantification of the blot image (**f**) (*n* = 3). **h** LPS-mediated p65 nuclear translocation was inhibited by transient α-tubulin overexpression. Data are presented as the mean ± SD (ns, not significant, *p* > 0.05; *p < 0.05; **p < 0.01; ***p < 0.001).

During activation of the NF-κB signaling pathway, IκB is phosphorylated and degraded by proteasomes. The released p65 from IκB is translocated to the nucleus, inducing the transcription of target genes for inflammatory responses^52^. Therefore, we monitored the translocation of p65 upon SB26019 treatment in J774A.1 and RAW264.7 murine macrophage cells stained with fluorescently labeled antibodies. Immunofluorescence patterns indicated that p65 resided mainly in the cytosol under basal conditions, whereas it predominantly translocated to the nucleus with LPS stimulation. Fluorescent colocalization analysis of p65 and the nucleus indicated that LPS increased p65 translocation to the nucleus from 40% to 80% in most J774A.1 and RAW264.7 cells (Fig. 3a, b and Supplementary Figs. 14, 15). However, treatment with colchicine-domain binders (nocodazole, colchicine, and SB26019) in the presence of LPS mitigated p65 nuclear translocation to <60% (Fig. 3b and Supplementary Fig. 15b).

To understand the inhibitory mechanism of p65 nuclear translocation by SB26019, the expression levels of p65, IκB, and tubulin monomers were monitored upon SB26019 treatment in murine macrophage cells. SB26019 induced IκB degradation in a time- and dose-dependent manner, which was inhibited by pretreatment with the proteasome inhibitor MG132 (Fig. 3c, d), indicating a possible dissociation of IκB from p65, followed by proteasome-mediated IκB degradation upon SB26019 treatment. Additionally, nuclear translocation of p65 was inhibited by SB26019 treatment despite an increase in phospho-IκB and a decrease in total IκB (Fig. 3e). To explain why the released p65 does not translocate to the nucleus, we hypothesized that the increased level of tubulin monomers induced by SB26019 facilitated its binding to p65. Consequently, IκB is released from p65 and degraded by the proteasome. Based on these observations, we postulated that the binding of α-tubulin monomers to p65 would block its nuclear translocation. To test this hypothesis, we monitored protein‒protein interactions among p65, IκB, and tubulin with immunoprecipitation. The interaction between p65 and α-tubulin was increased upon SB26019 treatment, as confirmed by immunoprecipitating either α-tubulin monomer or p65 (Fig. 3f, g and Supplementary Figs. 16, 17). When we further examined LPS-mediated p65 nuclear translocation upon α-tubulin overexpression, we observed that the increase in α-tubulin monomer levels perturbed LPS-mediated p65 nuclear translocation (Fig. 3h), demonstrating the role of the α-tubulin monomer in the inflammatory signaling pathway.

### SB26019 ameliorates neuroinflammation in vivo

**Fig. 4:**
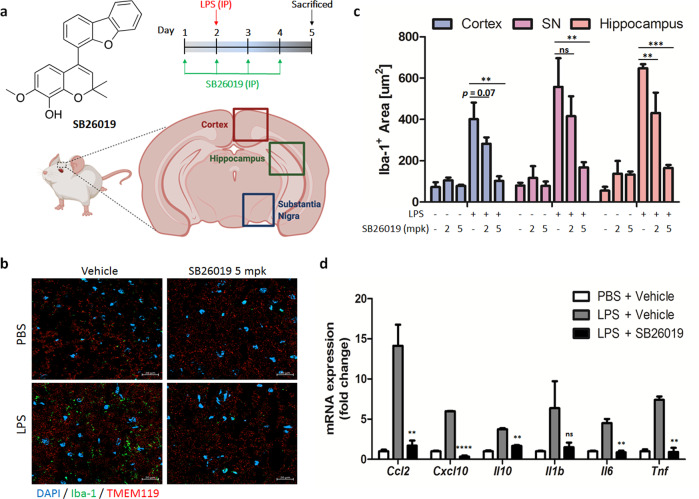
SB26019 ameliorates in vivo neuroinflammation in a mouse model. **a** Scheme of in vivo analysis. SB26019 (2 or 5 mg/kg body weight) or vehicle (distilled water containing 5% DMSO and 40% polyethylene glycol 400) was administered daily via intraperitoneal (IP) injection for 4 days. LPS (5 mg/kg) was administered via IP injection on Day 2 for a single challenge. All mice were sacrificed on Day 5. **b** After administration of SB26019 and LPS, the brains were harvested and dissected for fluorescent Iba-1 staining. SB26019 downregulated Iba-1 expression by inhibiting microglial activation (*n* = 3). **c** Quantification of the Iba-1-positive region of the mouse brain. LPS-induced neuroinflammation in all regions of the brain was downregulated by SB26019 in a dose-dependent manner. **d** After LPS and SB26019 administration, microglial cells in mouse brains were isolated and subjected to RNA extraction. qPCR showed that SB26019-modulated NF-κB activation in microglial cells (*n* = 3). Data are presented as the mean ± SD. (ns, not significant, *p* > 0.05; *p < 0.05; **p < 0.01; ***p < 0.001).

Finally, we tested the anti-neuroinflammatory efficacy of SB26019 in an LPS-induced murine model of neuroinflammation. Mice were treated with SB26019 via IP injection, *semel in die* (SID), for 4 days, followed by LPS injection once on Day 2. After sacrificing the mice on Day 5, their brains were cryosectioned for immunofluorescence imaging or minced to isolate primary microglia (Fig. 4a). Ionized calcium-binding adaptor protein-1 (Iba-1), an active microglia-specific marker, was stained to measure microglial activation. Since Iba-1 is involved in membrane ruffling, it is crucial for the morphological changes of microglia from a ramified to an activated state. Our immunohistochemistry data indicated that LPS treatment induced microglial activation, whereas SB26019 treatment ameliorated LPS-induced neuroinflammation without losing microglia in all brain regions (Fig. 4b and Supplementary Figs. 18, 19). Quantifying the Iba-1-positive region showed that SB26019 efficiently regulated microglial activation in all brain regions in a dose-dependent manner (Fig. 4c). For further validation, the transcription levels of cytokines and chemokines in the microglia of the mouse brain were analyzed; qPCR analysis showed that SB26019 efficiently inhibited the LPS-mediated upregulation of cytokines and chemokines in the mouse brain (Fig. 4d). The pharmacokinetic (PK) study showed that IP-injected SB26019 was absorbed quickly with a *T*_max_ of 0.17 h, a *C*_max_ of 1.20 μg/mL, and a half-life time (*t*_1/2_) of 3.57 h (Table 2).

**Table 2:** Pharmacokinetic (PK) analysis of SB26019.

Parameters	I.P., 5 mg/kg
*T*_max_ (h)	0.17 ± 0.00
*C*_max_ (μg/mL)	1.20 ± 0.26
*T*_1/2_ (h)	3.57 ± 0.62
AUC_t_ (μg h/mL)	1.77 ± 0.30
AUC_∞_ (μg h/mL)	1.79 ± 0.31
CL (L/h/kg)	NA
*V*_ss_ (L/kg)	NA
*F*_t_ (%)	NA

*NA* not applicable.

## Discussion

We discovered benzopyran-embedded anti-neuroinflammatory agents via phenotypic screening of our in-house 6000-membered pDOS library. One of the candidates for anti-neuroinflammation was previously reported as a tubulin inhibitor, compound **1** (Fig. 1a, b and Supplementary Fig. 1). Although colchicine has been used for treating gout^53^ and familial Mediterranean fever^54^ by inhibiting inflammation, tubulin modulators have not been explored as anti-neuroinflammatory agents. Moreover, there are only a few systematic reports about how tubulin-targeting agents show anti-inflammatory activities^38^. For these reasons, we selected compound **1** for further study to understand the relationship between neuroinflammation and tubulin modulation by utilizing it as a chemical probe even though compound **1** has moderate anti-neuroinflammatory activity. We first verified the specific binding site of compound **1** on tubulin to improve its efficacy. A photoaffinity-based competition assay revealed that compound **1** binds to the colchicine-binding domain of tubulin, which was further supported by in silico docking analysis (Fig. 1e, f and Supplementary Fig. 2). We improved the efficacy of compound **1** by conducting a SAR study and generated SB26019 as a lead compound (Table 1). The anti-neuroinflammatory activity of SB26019 was more potent than that of other known tubulin modulators, such as colchicine and nocodazole. Further biological studies showed that SB26019 efficiently inhibited proinflammatory cytokines, including TNF-α, IL-1β, IL-6, and chemokines (Fig. 2a, b and Supplementary Fig. 10).

To understand the mode of action of SB26019, we hypothesized that SB26019 might regulate neuroinflammation by controlling tubulin dynamics. After treatment with SB26019, we observed that tubulin monomer levels were increased (Fig. 2c, d). From this observation, we assumed that the increased level of tubulin monomer would contribute to the anti-neuroinflammatory activity of SB26019. Consequently, we overexpressed tubulin proteins in cells and monitored the levels of proinflammatory cytokines. Intriguingly, even a marginally elevated level of tubulin monomer downregulated downstream genes of NF-κB (Fig. 2e, f), supporting the critical role of the α-tubulin monomer itself in inflammatory signaling pathways.

To reveal the biological roles of tubulin monomers, we further monitored p65 nuclear translocation, a parameter for NF-κB activation, upon SB26019 treatment. SB26019 mitigated LPS-triggered nuclear translocation of p65 and inhibited the inflammatory signaling pathway (Fig. 3a, b and Supplementary Figs. 14, 15). Mechanistic studies revealed that SB26019 induced IκB degradation in a proteasome-dependent manner (Fig. 3c, d), indicating a possible dissociation of IκB from p65, followed by proteasome-mediated IκB degradation upon SB26019 treatment. Although SB26019 induced an increase in phospho-IκB and a decrease in total IκB (Fig. 3e), nuclear translocation of p65 was inhibited by SB26019. Considering that IκB phosphorylation normally leads to p65 nuclear translation, we cannot ignore the possibility of the off-target effect of SB26019. The upstream factor of IκB phosphorylation could be affected by SB62019, which needs to be validated in future research. Through monitoring protein‒protein interactions among p65, IκB, and tubulin with immunoprecipitation, we revealed that SB26019 treatment increased the interaction between p65 and α-tubulin (Fig. 3f, g and Supplementary Figs. 16, 17). We believe this interaction prohibits the nuclear translocation of p65 by perturbing the p65-mediated inflammatory pathway, which was also confirmed by α-tubulin overexpression (Fig. 3h). These observations suggest a new role for tubulin monomers in the inflammatory signaling pathway. Although the protein‒protein interaction between NF-κB and tubulin polymers was previously reported in cancer cells^55^, Panda and coworkers focused only on the role of tubulin polymers but not its monomer. Along with a previous report about tubulin monomers as c-Jun modulators^28^, our results demonstrated tubulin monomers as protein modulators beyond their cellular organizational function in neuroinflammatory events. We also confirmed that SB26019 regulated neuroinflammation by suppressing microglial activation in vivo (Fig. 4). Collectively, our findings demonstrated that colchicine-binding site-specific modulation of tubulin could be a potential strategy for treating neurodegenerative diseases.

Although our results showed the effective anti-inflammatory activity of SB26019 in an LPS-mediated neuroinflammation model, long-term treatment with tubulin modulators might induce unwanted side effects due to their inherent cytotoxicity, which could be a significant limitation for their clinical applications in neurodegenerative disease treatment. Subsequent research will explore the proper dosage and toxicity of tubulin modulators for the long-term treatment of neuroinflammation. Combination therapy could be another possibility for effective anti-neuroinflammation treatment; cotreatment of a lower dosage of tubulin modulators with other anti-neuroinflammatory agents having different modes of action could be pursued in future work.

Even though tubulin modulators have been primarily considered anticancer agents due to their cytotoxicity, colchicine was approved for the treatment of gout^56^ and familial Mediterranean fever^54^, as previously mentioned. Colchicine was also tested as a therapeutic agent for Behçet’s disease^28^ and the prevention of atrial fibrillation after cardiac surgery^29^. Moreover, there is growing attention on colchicine as an anti-inflammatory reagent against COVID-19^30–32^ and cardiovascular disease^33–36^. Considering this recent revisit of tubulin modulators for various inflammatory diseases^31,32,34,36,37^ and hepatocyte-mediated myeloid cell inactivation^38^, neuroinflammation could be a new territory to be explored using colchicine-binding site-specific tubulin modulators. Therefore, discovering various types of tubulin modulators is essential for the efficient control of neuroinflammation to overcome the complexity of neuroinflammatory processes and the poor drug transport to brains due to the blood‒brain barrier, leading to the development of tubulin-targeting NCEs for the treatment of neurodegenerative diseases. We believe our findings could pave the way for colchicine-binding site-specific tubulin modulators as anti-neuroinflammatory agents.

## Supplementary information

supplementary information

## Data Availability

The main data supporting the results of this study are available within the paper and its supplementary information. Raw datasets generated and analyzed during the study are available from the corresponding authors upon reasonable request.
