# Supplementary material for: Phenotype-based screening rediscovered benzopyran-embedded microtubule inhibitors as anti-neuroinflammatory agents by modulating the tubulin–p65 interaction
Source: Exp Mol Med. 2022 Dec 12;54(12):2200–9. doi: 10.1038/s12276-022-00903-z (PMC9743128; doi:10.1038/s12276-022-00903-z)
Supplement: Supplementary file 1 — supplementary information [file 12276_2022_903_MOESM1_ESM.pdf]

Table of contents

Supplementary materials and methods for biological experiments 2

Supplementary figures

Supplementary Fig. 1 | Phenotype-based screening. 4

Supplementary Fig. 2 | Binding sites of known tubulin inhibitors. 5

Supplementary Fig. 3 | CETSA and photoaffinity-based competition assay. 6

Supplementary Fig. 4 | Tubulin polymerization inhibition assay. 7

Supplementary Fig. 5 | Dose-dependent NO release inhibitory activity and cytotoxicity of the focused library compounds. 8

Supplementary Fig. 6 | Dose-dependent cytotoxicity of compound 8. 9

Supplementary Fig. 7 | Correlation between anti-inflammatory effect and absolute C-Docker Interaction Energy. 9

Supplementary Fig. 8 | Anti-inflammatory effects of SB26019 compensated by Taxol. 10

Supplementary Fig. 9 | SB26019-mediated NF-κB inhibition and AP-1 activation compensated by Taxol. 10

Supplementary Fig. 10 | SB26019-mediated mRNA levels of NF-κB downstream genes in BV-2. 11

Supplementary Fig. 11 | Transient overexpression of α-tubulin in RAW264.7. 12

Supplementary Fig. 12 | SB26019 treatment or transient α-tubulin overexpression modulated mRNA levels of NF-κB downstream genes in RAW264.7. 12

Supplementary Fig. 13 | SB26019-mediated mitotic arrest at various time points. 13

Supplementary Fig. 14 | Immunofluorescent staining of p65 in J774A.1. 14

Supplementary Fig. 15 | Immunofluorescent staining of p65 in RAW264.7. 15

Supplementary Fig. 16 | Tubulin-p65 interaction in RAW264.7. 16

Supplementary Fig. 17 | α-Tubulin monomer-p65 interaction in RAW264.7. 16

Supplementary Fig. 18 | Immunofluorescent staining of the brain regions with TMEM119. 17

Supplementary Fig. 19 | Immunofluorescent staining of the brain regions. 18

Supplementary Tables

Supplementary Table 1. Initial structure-activity relationship study of benzopyran-embedded compounds. 19

Supplementary Table 2. Tubulin polymerization inhibition of the focused library. 20

Supplementary Table 3. DNA sequences of qPCR primers. 21

Supplementary materials and methods for chemical synthesis 22

Synthesis and characterization of compounds. 23

^{1}H and ^{13}C NMR spectra. 27

Reference 35

SUPPLEMENTARY MATERIALS AND METHODS FOR BIOLOGICAL EXPERIMENTS

Cell Culture

BV-2 (a murine microglial cell line) and RAW 264.7 (a murine macrophage cell line) were obtained from American Type Culture Collection and cultured in Dulbecco's modified Eagle's medium (DMEM) (Gibco; #11995-073) supplemented with 1% (v/v) antibiotic-antimycotic solution (Gibco; #15240-062) and heat-inactivated 5% (v/v) fetal bovine serum (FBS) (Gibco; #16000-044). J774A.1 (a murine macrophage cell line) was obtained from Korean Cell Line Bank and cultured in RPMI medium with 25 mM HEPES (*N*-2-hydroxyethylpiperzaine-*N'*-2-ethanesulfonic acid) (Gibco; #22400-089) supplemented with 1% (v/v) antibiotic-antimycotic solution and heat-inactivated 10% FBS (v/v). THP-1 (a human monocyte cell) was obtained from Korean Cell Line Bank and cultured in the same conditioned media with J774A.1 supplemented with 0.1% (v/v) 2-mercaptoethanol (Gibco; # 21-985-023). HEK293T (a human embryonic kidney) cell with NF-κB SEAP reporter (Invivogen; hkb-mmcl) was cultured in DMEM (Gibco; #11995-073) supplemented with 1% (v/v) antibiotic-antimycotic solution (Gibco; #15240-062) and heat-inactivated 10% (v/v) FBS (Gibco; #16000-044). All types of cells were maintained in 100-mm cell culture dishes in a humidified atmosphere of a 5% CO_2 incubator at 37 °C. HEK293T cells were cultured every 2 days using trypsin (Gibco; #12605-010). All murine immune cells were detached by pipetting without using trypsin and cultured every 2 days.

Transient Tubulin and GFP Overexpression

α-Tubulin plasmid (Origene; RC208669) was transiently transfected to RAW264.7 cells via electroporation with SF Cell Line 4D-Nucleofector^{TM} X Kit (Lonza; #V4XC-2024). α-Tubulin overexpression was confirmed with western blot using anti-α-tubulin and anti-FLAG primary antibodies. α-Tubulin plasmid (Origene; RC208669) and GFP plasmid were transiently transfected to HEK293T cells using LTX plus (Invitrogen; 15338100) and opti-MEM (Gibco; 31985070) according to the manufacturer's protocol for 1 and 2 days. α-Tubulin and GFP overexpression was confirmed with western blot using anti-α-tubulin and anti-GFP primary antibodies, respectively.

Subcellular fractionation

Nucleus and cytosol fraction of cells were extracted by NE-PER™ Nuclear and Cytoplasmic Extraction Reagents (Thermo; 78835) via the manufacturer's protocol. Briefly, cells were re-suspended in CER I buffer. After 15 sec of vortexing, cells were incubated for 10 min at 4 °C. After 5 sec of vortexing, CER II buffer was added to cell lysates and incubated for 1 min, followed by 5 sec of vortexing and centrifugation at 13,000 g, 4 °C for 5 min. Supernatant was immediately transferred to a clean pre-chilled e-tube. Insoluble pellet fraction was re-suspended in NER buffer and incubated for 10 min at 4 °C followed by 15 sec of vortexing for four times. Clear nuclear fraction was transferred to a clean pre-chilled e-tube after centrifugation at 13,000 g, 4 °C for 10 min. The protein concentration of the nucleus and cytosol fraction was measured by BCA protein assay kit (Thermo; #23225).

Reporter Gene Assay

NF-κB SEAP reporter HEK293T cell (Invivogen; hkb-mmcl) were seeded 10,000 cells/well at white 96-well plate (Falcon; 353296). HEK293 cells were transfected with a 1 to 1 ratio of 3xAP1pGL3 (Addgene; #40342) and pRL-TK (Promega; E2241) mixture using LTX plus (Invitrogen; 15338100) and opti-MEM (Gibco; 31985070) according to the manufacturer's protocol. After 24-h compound treatment or the overexpression of α-tubulin or GFP, 40 μl of the medium was transferred to a 96-well plate and mixed with 180 μl of quanti-blue solution (Invitrogen; rep-qbs) for the measurement of NF-κB activation. We measured the absorbance at 655 nm after 2-h incubation at 37 °C. To measure the AP-1 activation, cells were washed with PBS and lysed with 20 μl of 1× passive lysis buffer for 15 min. Luciferase signals were measured by dual-luciferase reporter assay system (Progema; E1980) using a microplate reader (BioTek; Synergy HTX). Expression levels of SEAP and AP-1 were normalized by renilla luciferase signal.

Flow Cytometry Analysis

J774A.1 cells were treated with each compound for the designated time and dose. The cells were trypsinized and re-suspended in PBS containing 2% FBS (Gibco; #16000-044). To measure cell cycle arrest, the same number of the re-suspended cells was stained with propidium iodide (PI) (Abcam; ab14085) according to the manufacturer’s protocol. The stained cells were subjected for flow cytometry analysis using FACSAria II (BD).

Supplementary Figures

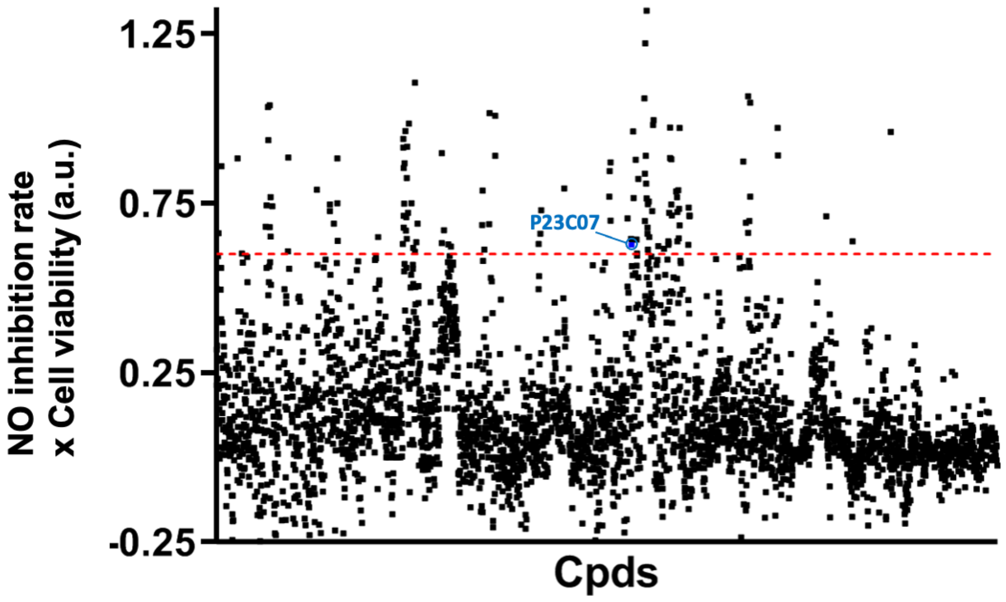

Supplementary Fig. 1. Phenotype-based screening of our in-house 6,000-membered pDOS library via monitoring cellular nitric oxide (NO) release in BV-2 murine microglial cells. To find the hit compounds with high NO inhibition activity and low cytotoxicity, we performed NO release assay and cytotoxicity test simultaneously. NO inhibition rate of compounds was multiplied by cell viability of each compound. The top 5% compounds (above the red dotted line) were selected as initial hit compounds. Among the initial hit compounds, we narrowed the list of compounds down by removing the compounds with structural similarities or by including compounds with validated protein targets. Considering tubulin modulators have not been explored as anti-neuroinflammatory agents so far, we have finally selected P23C07 (Compound **1**) for further study to understand the relationship between neuroinflammation and tubulin modulation by hiring it as a chemical probe despite its moderate anti-neuroinflammatory activity.

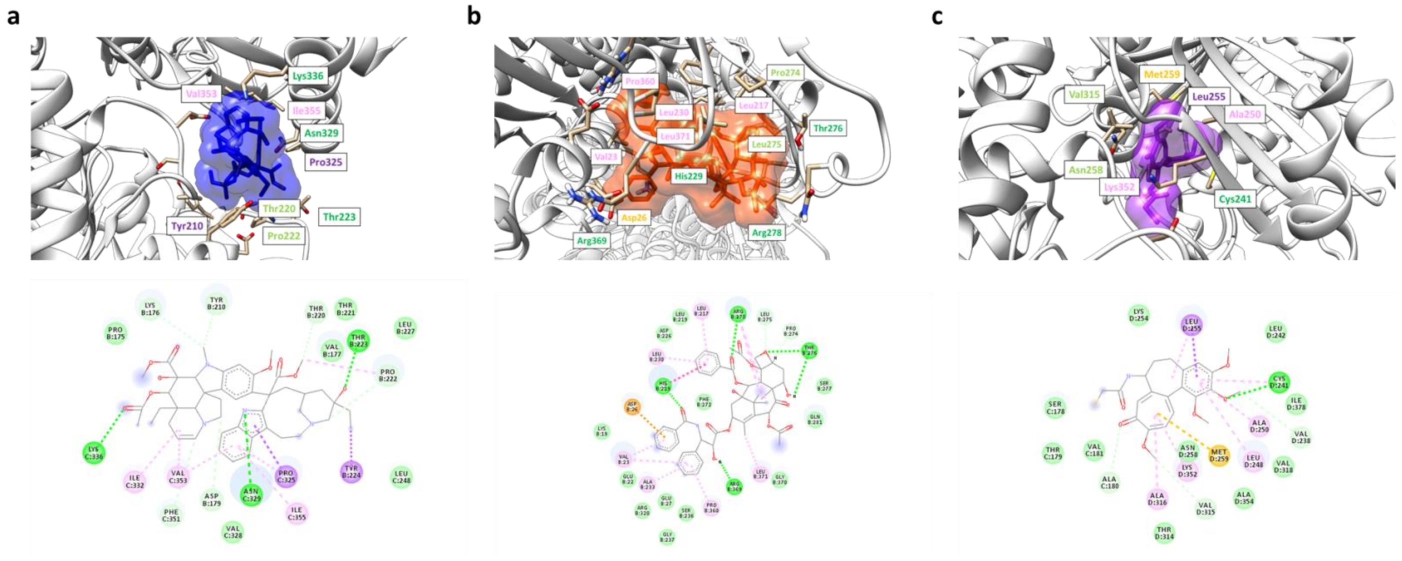

Supplementary Fig. 2. Binding sites of known tubulin inhibitors pertaining to Figure 1c, (a) vinblastine (blue), (b) taxol (Orange), and (c) colchicine (purple).

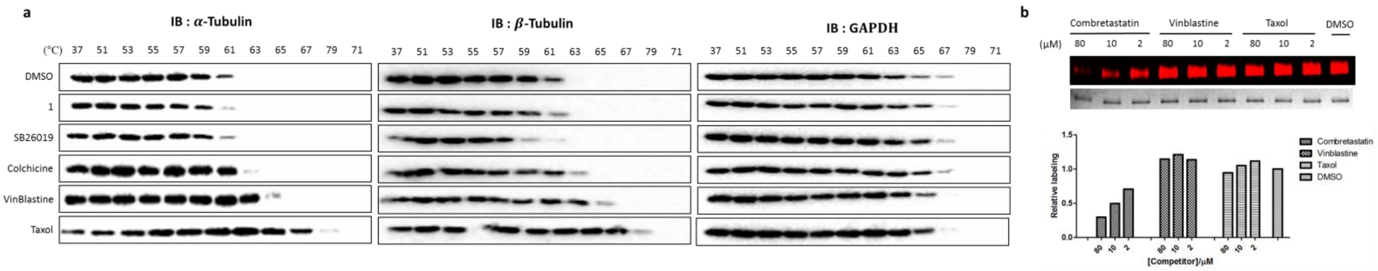

Supplementary Fig. 3. Tubulin-binding site of compound **1** was identified with photoaffinity-based competition assay approach. (a) Three known tubulin binders (colchicine, vinblastine, and taxol) induced the resistance of tubulin protein against heat denaturation in cellular thermal shifts assay (CETSA), but **1** did not. (b) Photoaffinity-based competition assay verified that compound **1** bound to colchicine-binding sites. Tubulin protein (10 μM) was incubated with target ID probe of **1** (5 μM) in the absence or presence of known tubulin binders for 75 min. After 365-nm UV irradiation for photo-crosslinking, Cy5-azide was conjugated with click reaction. Similar to colchicine (Fig. 1e), combretastatin (a tubulin binder sharing the colchicine-binding site) also showed a dose-dependent competition with target ID probe of **1**, which confirmed the binding site of **1**.

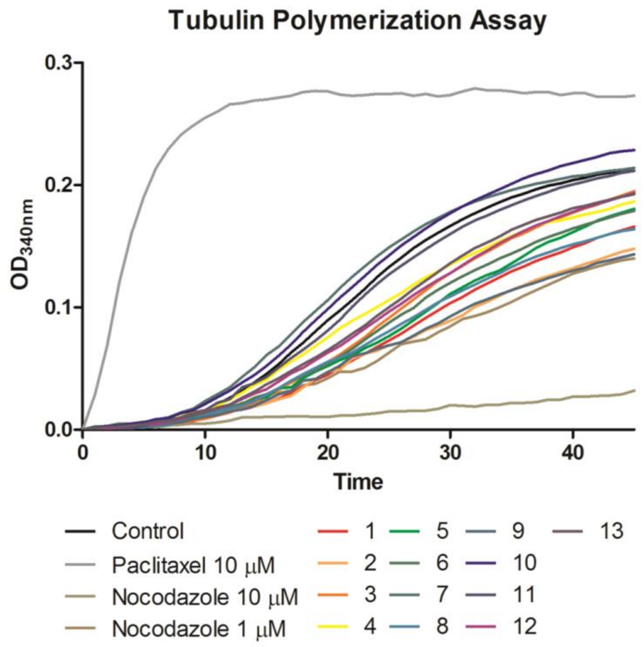

Supplementary Fig. 4. Tubulin polymerization inhibition by compounds **1–13** (1 μM). In the presence or absence of compounds, tubulin was incubated to polymerize at room temperature. Absorbance at 340 nm was measured in a time-dependent manner for monitoring the rate of tubulin polymerization.

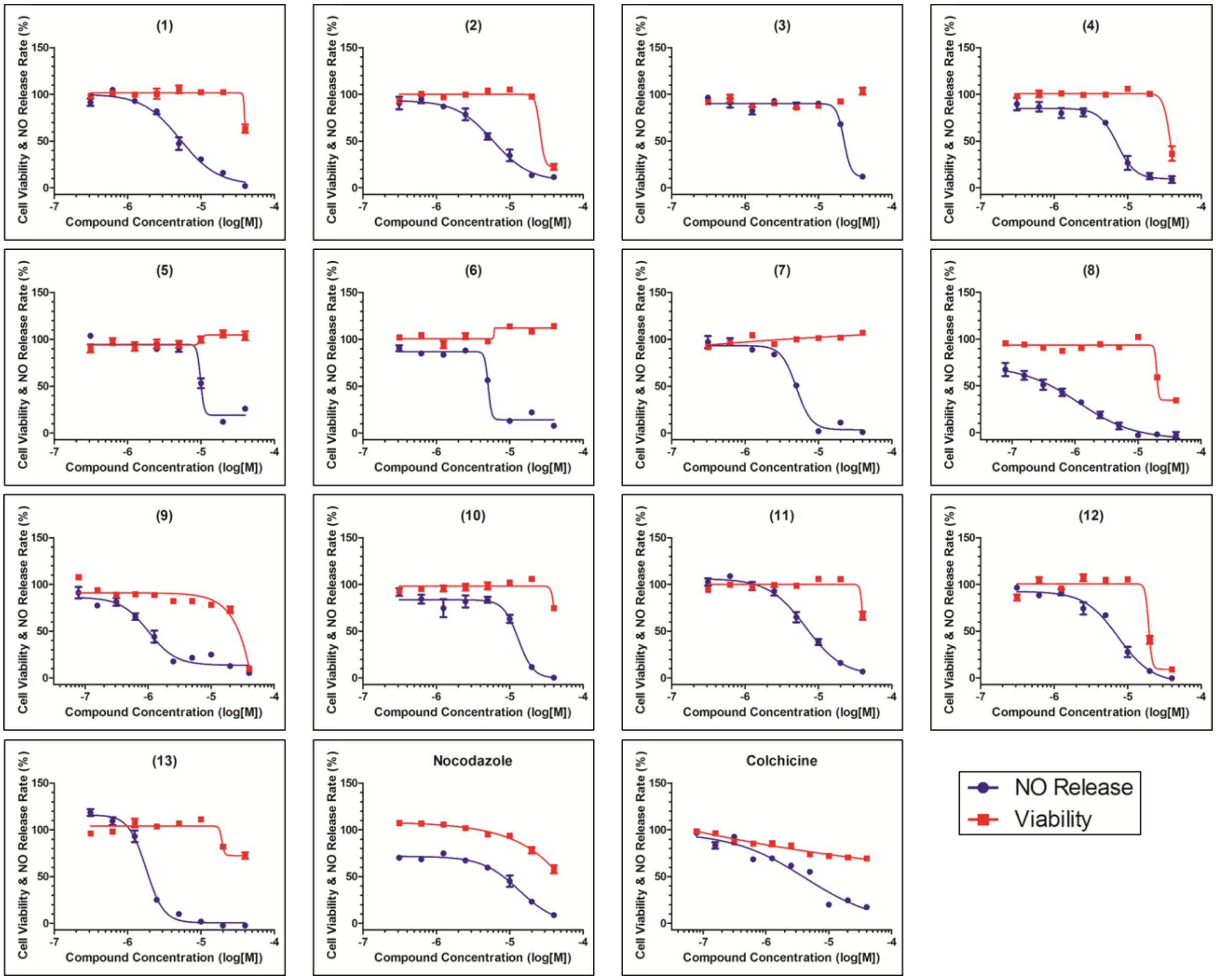

Supplementary Fig. 5. Dose-dependent NO release inhibitory activity and cytotoxicity of the focused library compounds (n=6). These data are summarized in Table 1. Data are presented as the mean ± SD (standard deviation).

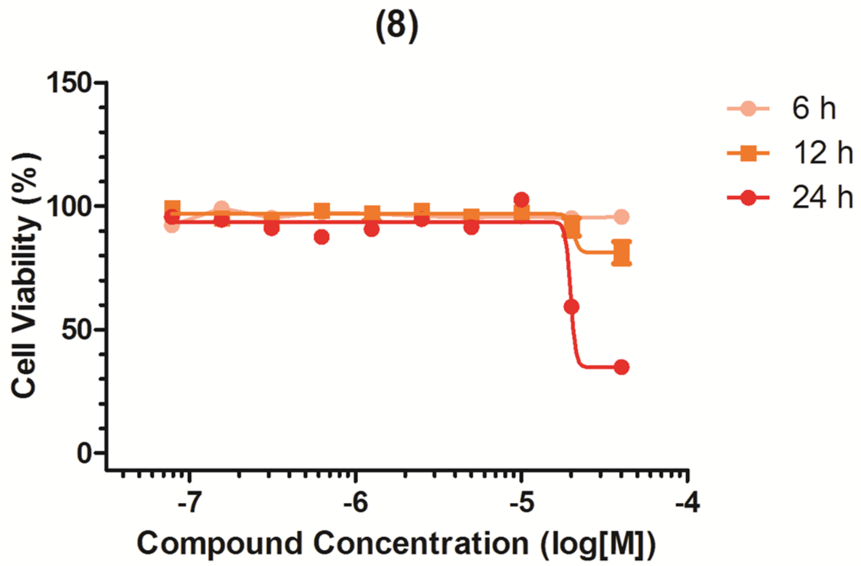

Supplementary Fig. 6. Cell viability curve for compound **8** at various time point in BV-2, microglial cell (n=6). Data are presented as the mean ± SD.

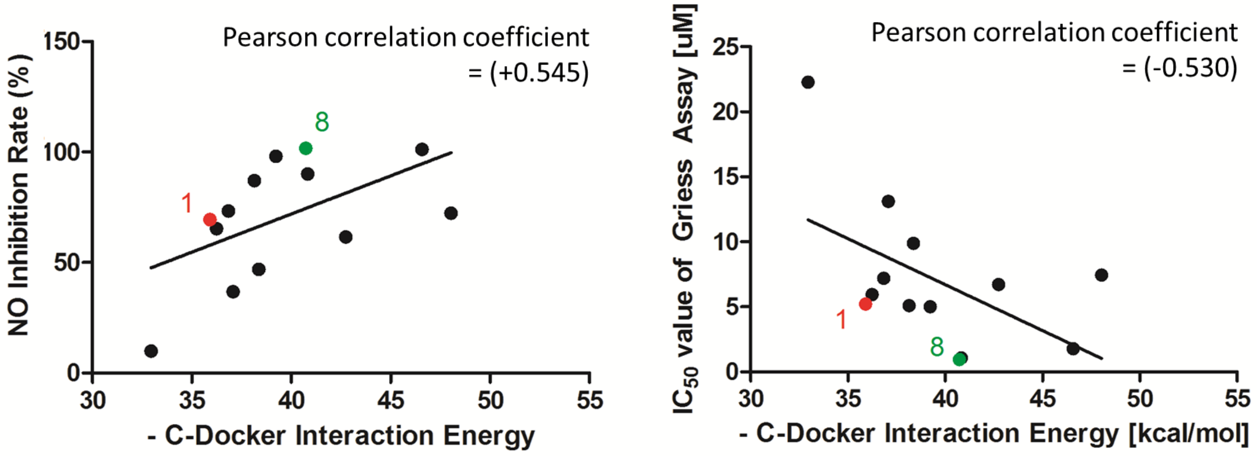

Supplementary Fig. 7. Correlation between anti-inflammatory effect and absolute C-Docker Interaction Energy. The correlations showed reasonable Pearson correlation coefficient values.

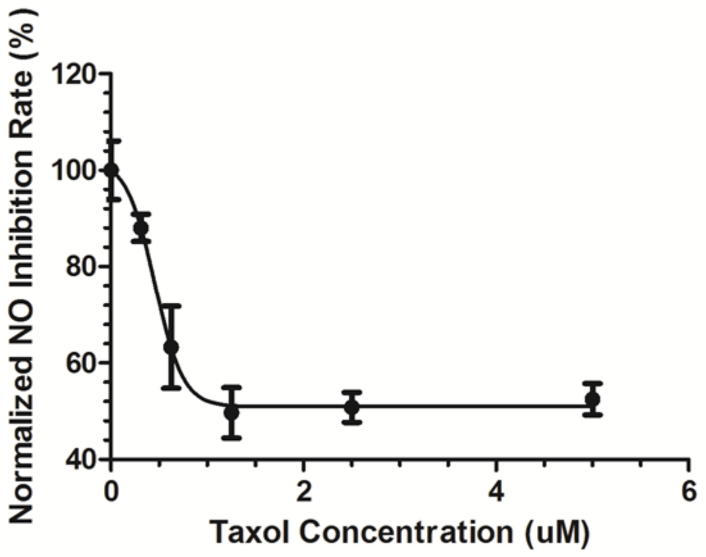

Supplementary Fig. 8. Anti-inflammatory effects of SB26019 (5 μM) were compensated by tubulin stabilizer (taxol) in a dose-dependent manner (n=6). Data are presented as the mean ± SD.

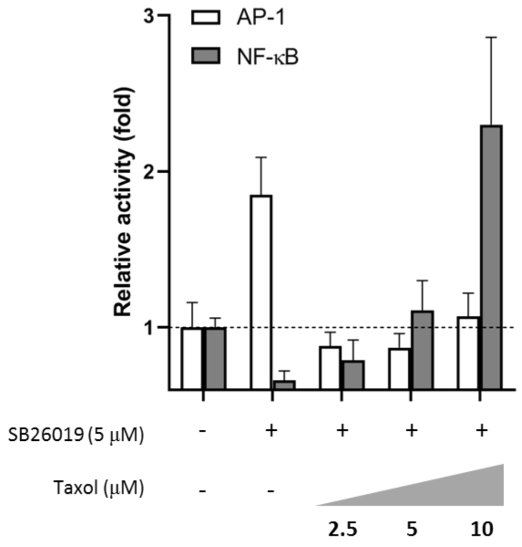

Supplementary Fig. 9. SB26019-mediated NF-κB inhibition and AP-1 activation were compensated by taxol treatment in a dose-dependent manner (n=6). NF-κB, a master regulator of inflammation, was inhibited by SB26019, but re-activated by taxol. Considering the previous report about AP-1 activation by tubulin monomers,[1] we also observed the activation of AP-1 by SB26019-mediated increment of tubulin monomers. Data are presented as the mean ± SD.

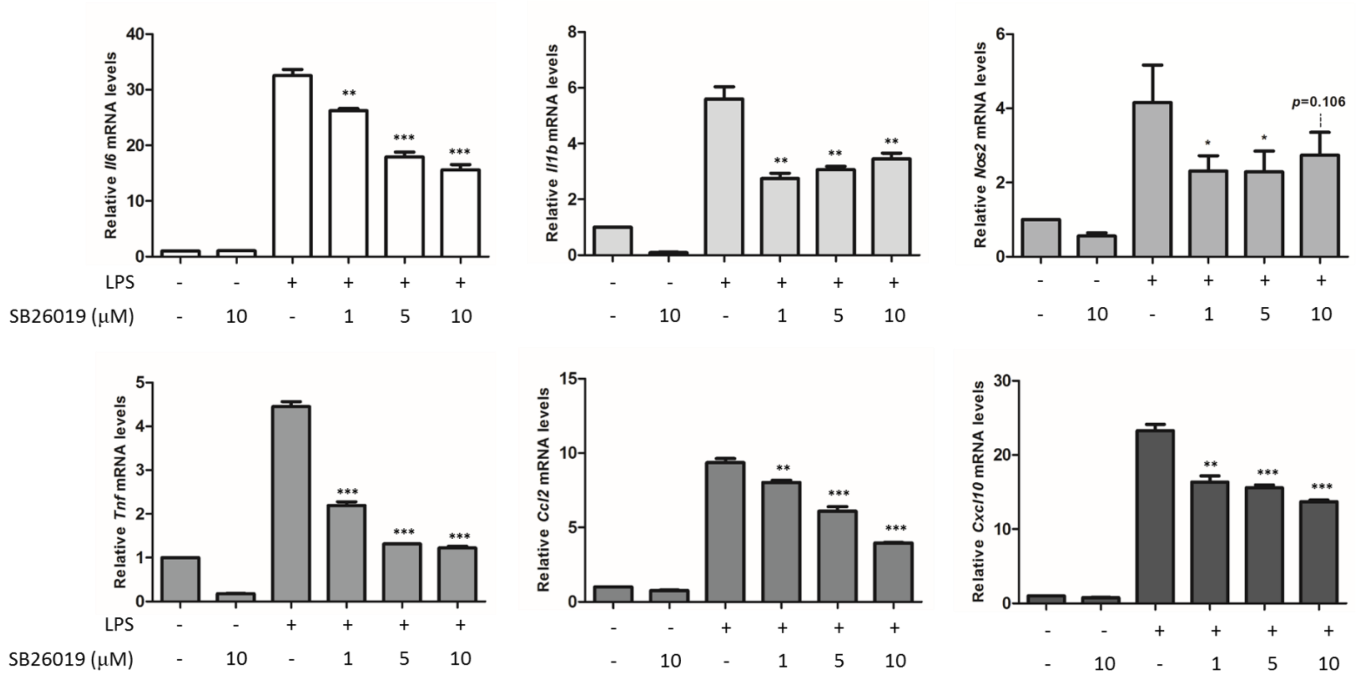

Supplementary Fig. 10. SB26019 modulated mRNA levels of NF-κB downstream genes in BV-2 murine microglia cells in a dose-dependent manner (n=3). Full data set of Figure 2a. Data are presented as the mean ± SD (ns, not significant, $p > 0.05$; *, $p < 0.05$; **, $p < 0.01$; ***, $p < 0.001$).

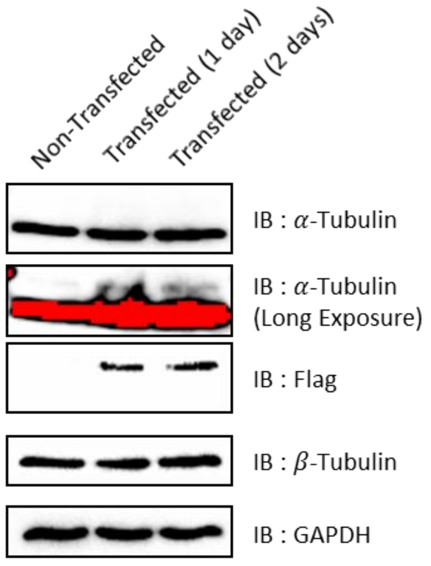

Supplementary Fig. 11. Transient overexpression of α-tubulin in RAW264.7 murine macrophage cells. Flag-tagged α-tubulin was overexpressed to monitor the anti-inflammatory effect mediated by tubulin monomers.

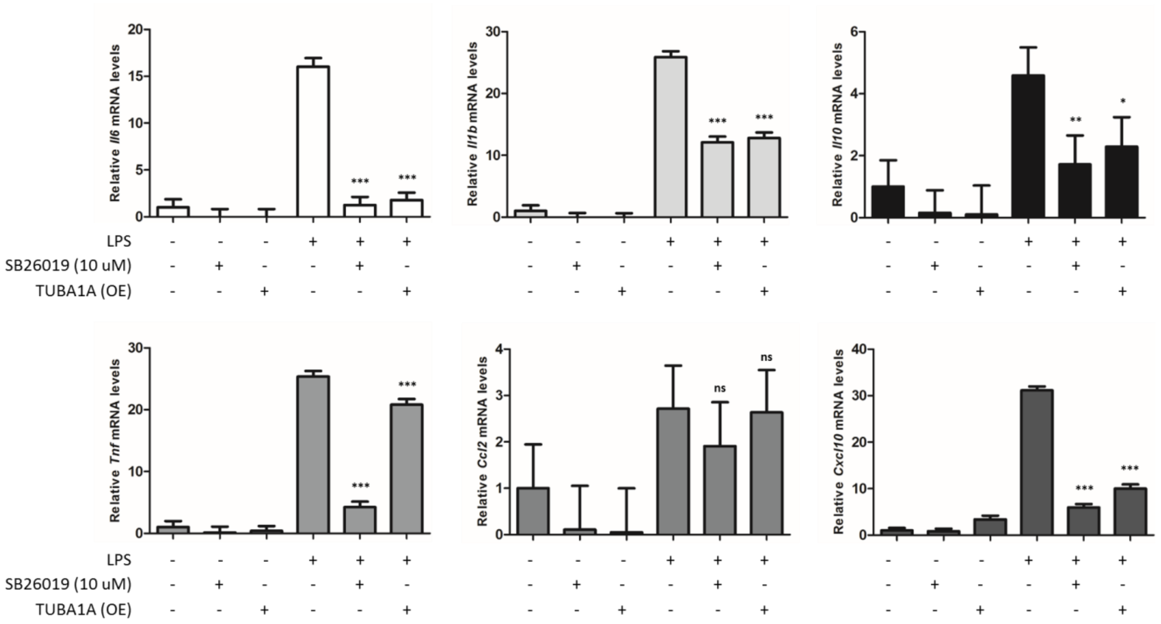

Supplementary Fig. 12. SB26019 treatment or transient α-tubulin overexpression modulated mRNA levels of NF-κB downstream genes in RAW264.7 murine macrophage cells (n=3). Full data set of Figure 2e. Data are presented as the mean ± SD (ns, not significant, $p > 0.05$; *, $p < 0.05$; **, $p < 0.01$; ***, $p < 0.001$).

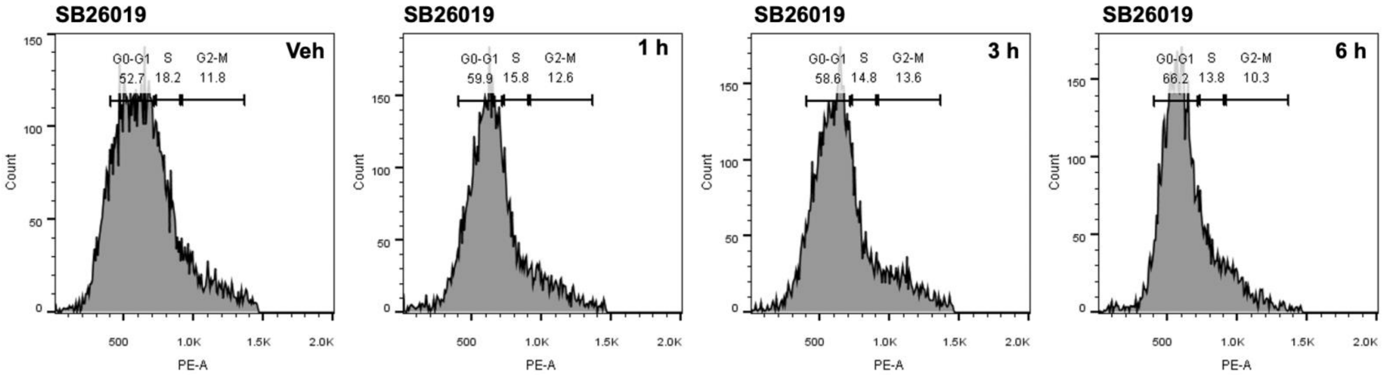

Supplementary Fig. 13. 20 μM of SB26019 treatment didn't show any mitotic arrest within 6 h confirmed by flow cytometry in J774A.1 murine macrophage cell.

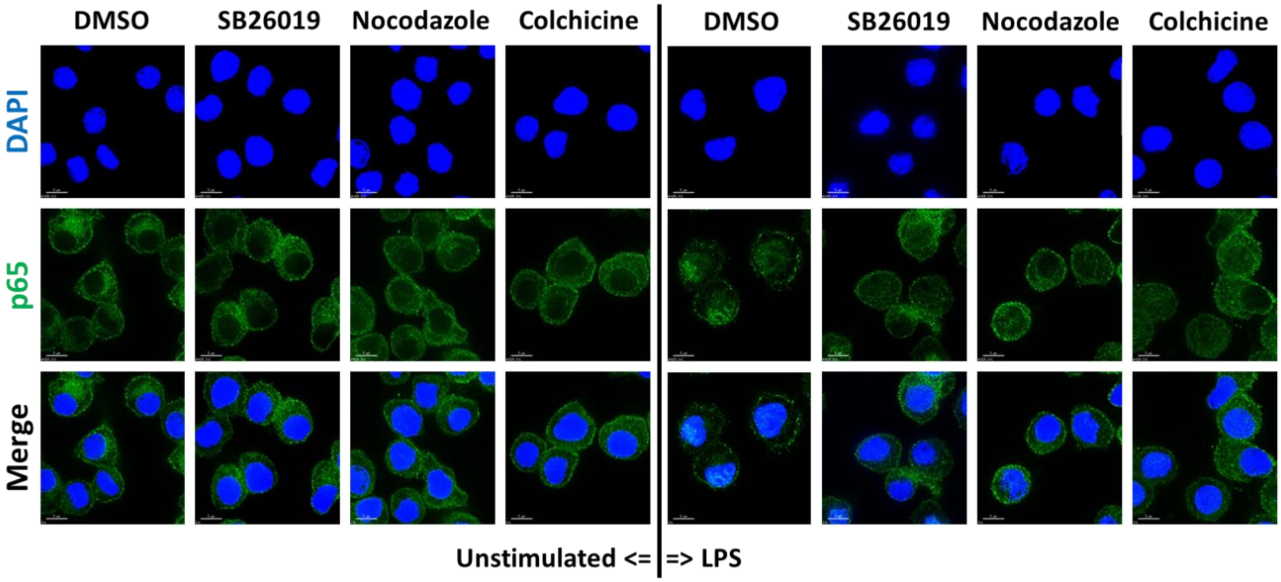

Supplementary Fig. 14. Immunofluorescent staining of p65 images in J774A.1 murine macrophage cells. Full data set of Figure 3a (The scale bar represents 5 μm).

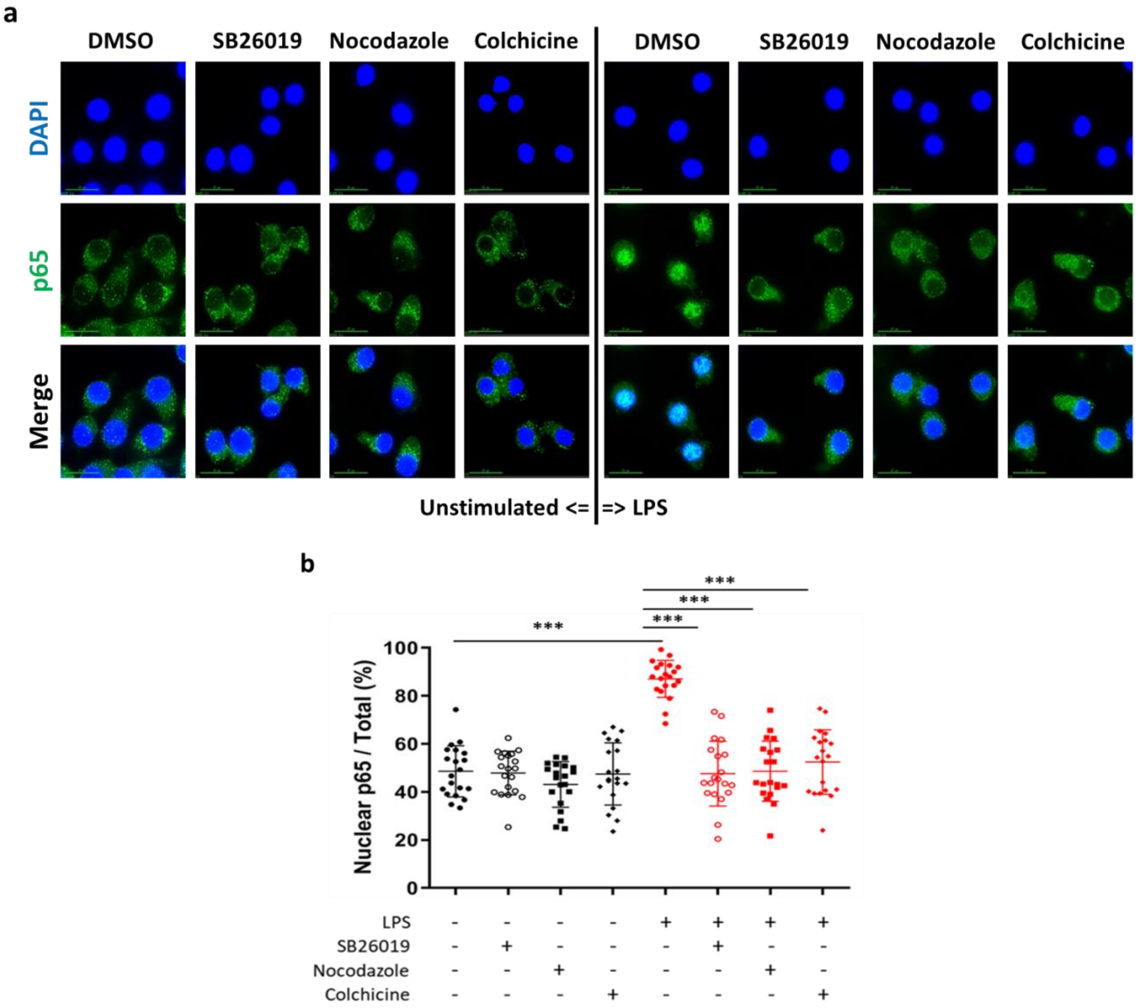

Supplementary Fig. 15. (a) Immunofluorescent staining of p65 images in RAW264.7 murine macrophage cells. (b) Quantification of fluorescence images (a) (The scale bar represents 15 μm).

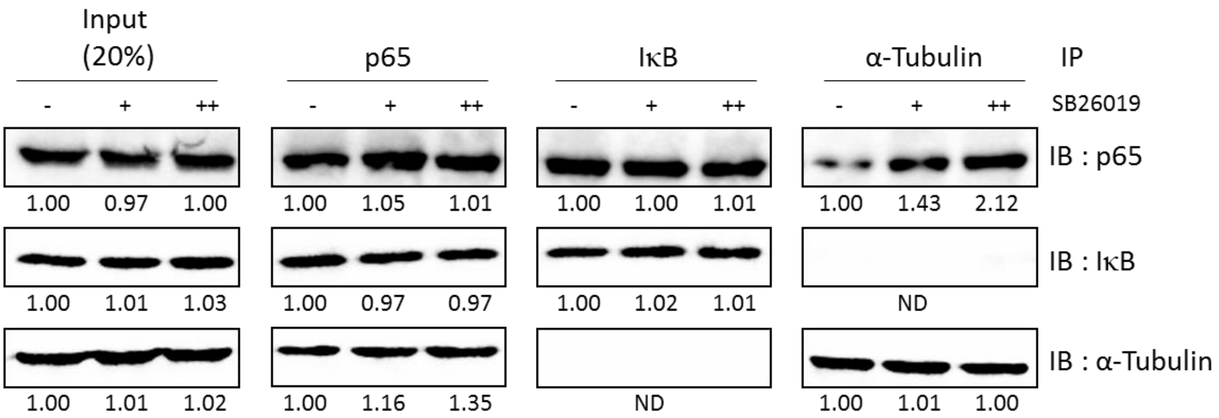

Supplementary Fig. 16. Tubulin-p65 interaction increased after 1-h treatment of SB26019 (+; 10 μM, ++; 20 μM) in RAW264.7 murine macrophage. Increased levels of tubulin bound to p65, generating tubulin–p65 complex.

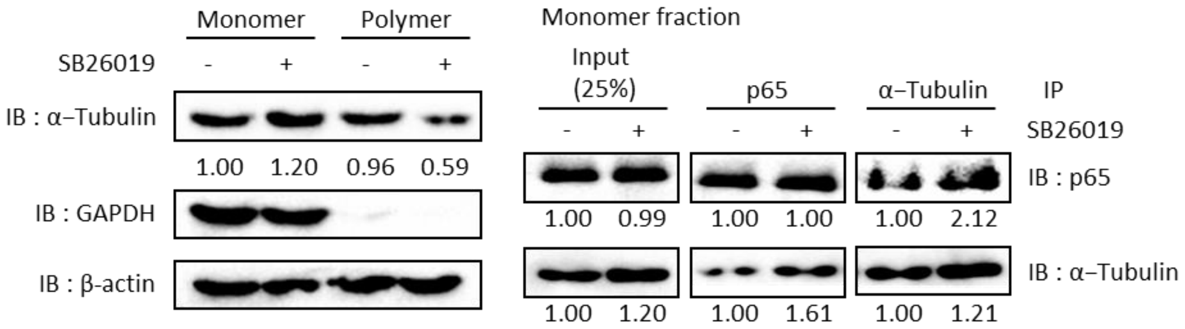

Supplementary Fig. 17. α-Tubulin monomer-p65 interaction increased after 1-h treatment of SB26019 (10 μM) in RAW264.7 murine macrophage.

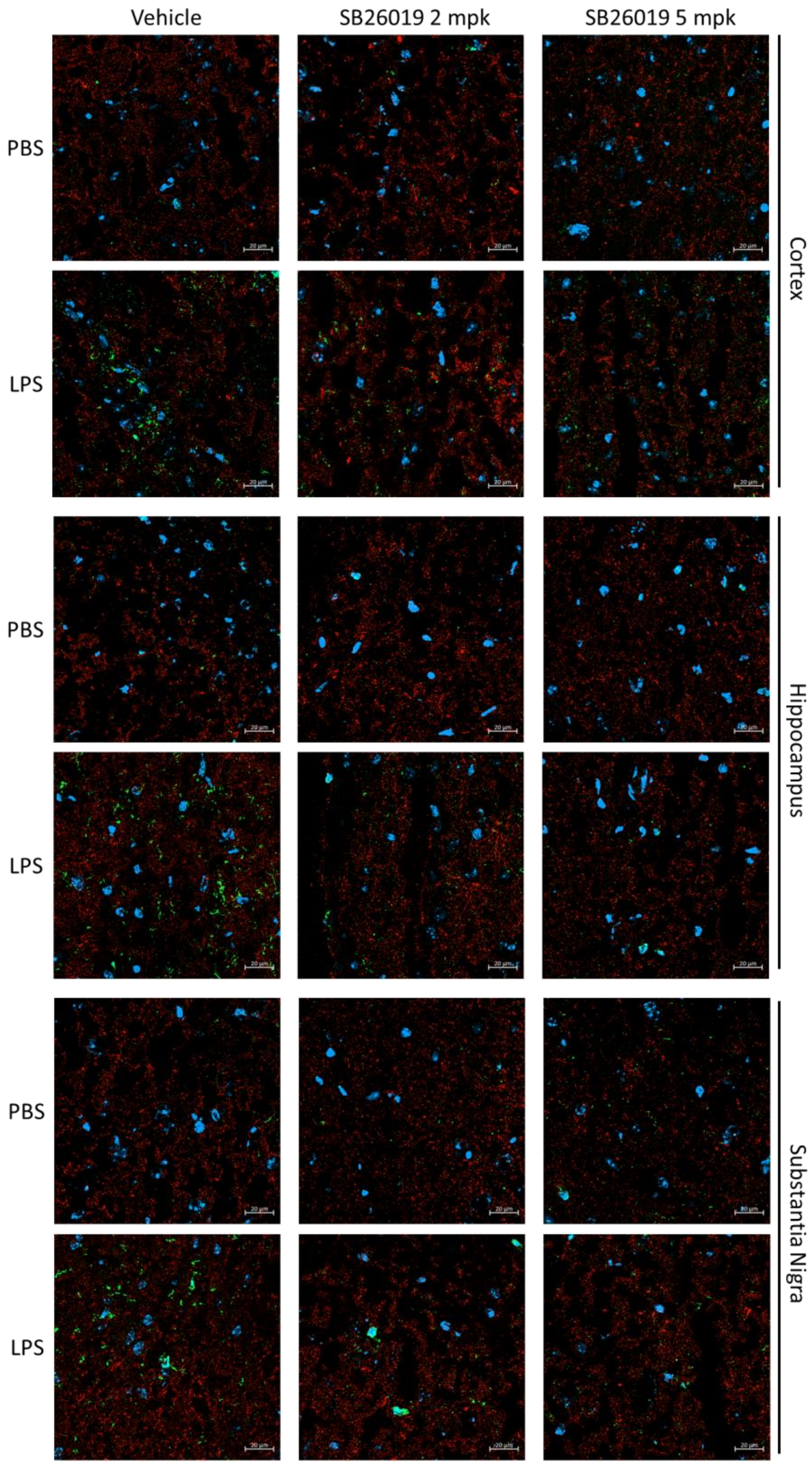

Supplementary Fig. 18. Immunofluorescent staining images of the various brain regions (cortex, hippocampus, and substantia nigra). Full data set of Figure 4b. SB26019 ameliorates neuroinflammation *in vivo* mouse model without loss of microglia. (blue, DAPI staining; green, Iba-1 staining; red, TMEM119 staining; the scale bar represents 20 μm; TMEM119 is a macroglia marker protein)

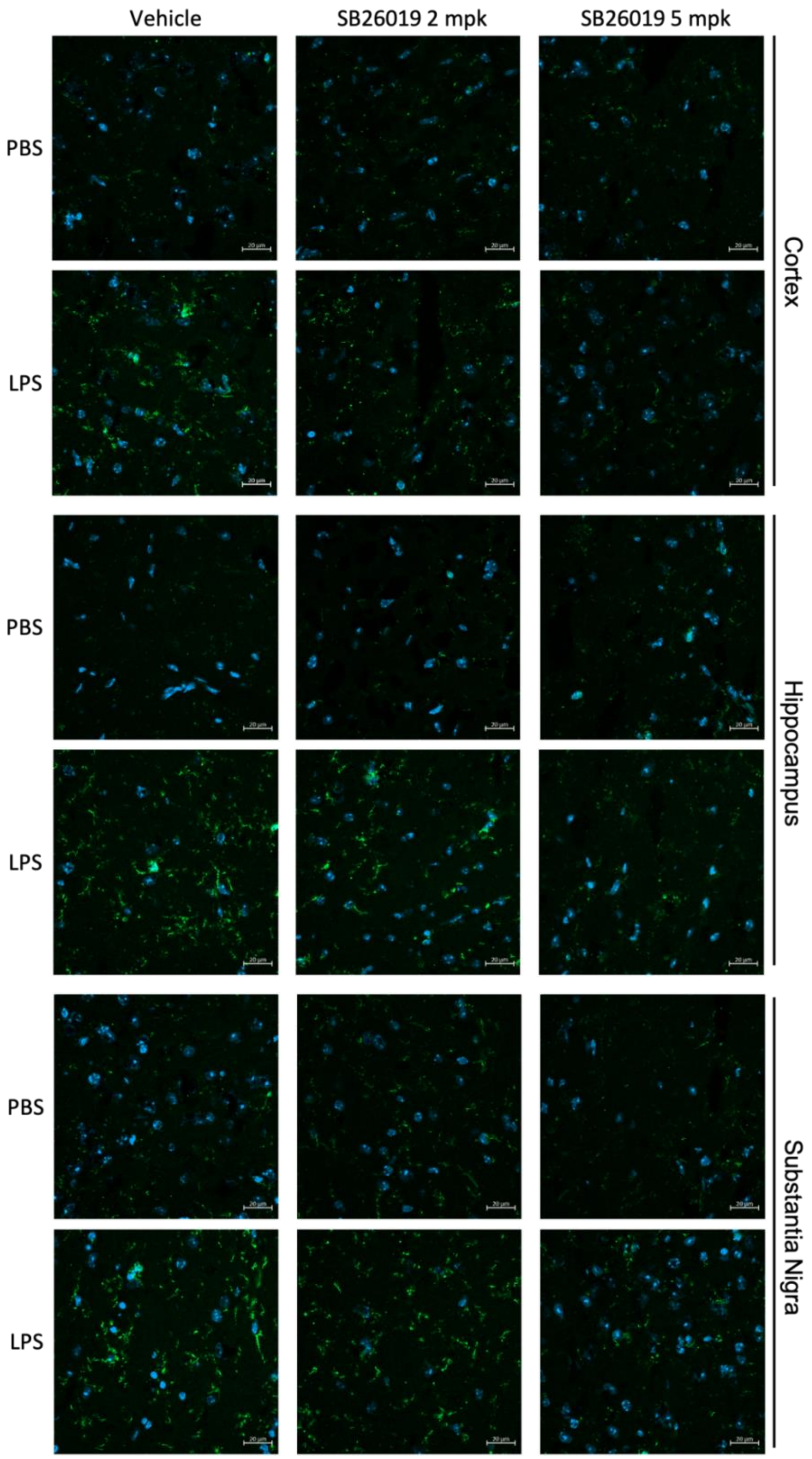

Supplementary Fig. 19. Immunofluorescent staining images of the brain regions. (blue, DAPI staining; green, Iba-1 staining; the scale bar represents 20 μm; Iba-1 is an active microglia-specific marker).

Supplementary Table 1. Initial structure-activity relationship study of benzopyran-embedded compounds**.**

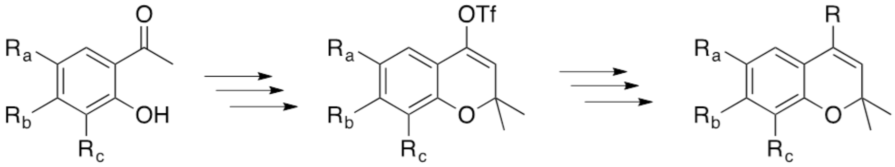

Cpd.	R	R_a	R_b	R_c	% NO inh. at 10 μM
**1**	4-chloro-2-methylphenyl	H	methoxy	hydroxy	80.9
**1a**	4-chloro-2-methylphenyl	H	hydroxy	H	17.3
**1b**	4-chloro-2-methylphenyl	H	methoxy	methoxy	12.9
**1c**	4-chloro-2-methylphenyl	Cl	H	3-hydroxylphenyl	< 0

Supplementary Table 2. Tubulin polymerization inhibition of the focused library. Tubulin was incubated with each compound (1 μM) and the tubulin polymerization inhibition was monitored. V_{max} was calculated from tubulin polymer generation curve data of Supplementary Fig. 3**.**

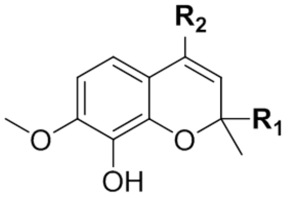

Cpd.	R_1	R_2	Vmax (mOD/min)
Vehicle			9.5
**1**	methyl	4-chloro-2-methylphenyl	6.5
**2**	methyl	4-fluoro-2-methylphenyl	9
**3**	methyl	4-fluorophenyl	8.5
**4**	methyl	2-methylphenyl	8.5
**5**	methyl	2,5-dimethylphenyl	7.5
**6**	methyl	2-methoxyphenyl	8.5
**7**	methyl	quinolin-8-yl	10
**8**	methyl	dibenzofuran-4-yl	6
**9**	methyl	dibenzothiophen-4-yl	8.5
**10**	methyl	thianthren-1-yl	10.5
**11**	methyl	1,1-biphenyl-3-yl	10.5
**12**	ethyl propionate	4-chloro-2-methylphenyl	7
**13**	ethyl propionate	dibenzofuran-4-yl	8
Nocodazole (1 μM)			7
Nocodazole (10 μM)			3
Taxol (10 μM)			51.5

Supplementary Table 3. DNA sequences of qPCR primers.

Genes		Primer Sequences
Cytokines		
	Il6	F : 5'- TCC AGT TGC CTT CTT GGG AC -3'<br>R : 5'- GTA CTC CAG AAG ACC AGA GG -3'
	Il1b	F : 5'- AAG TTG ACG GAC CCC AAA AGA T -3'<br>R : 5'- TGT TGA TGT GCT GCT GCG A -3'
	Il10	F : 5'- ATA ACT GCA CCC ACT TCC CA -3'<br>R : 5'- GGG CAT CAC TTC TAC CAG GT -3'
	Nos2	F : 5'- GCC ACC AAC AAT GGC AAC A -3'<br>R : 5'- CGT ACC GGA TGA GCT GTG AAT T -3'
	Tnf	F : 5'- ATG GCC TCC CTC TCA TCA GTT C -3'<br>R : 5'- TTG GTG GTT TGC TAC GAC GTG -3'
Chemokines		
	Ccl2	F : 5'-TCA GCC AGA TGC AGT TAA CG-3'<br>R : 5'-GAT CCT CTT GTA GCT CTC CAG C-3'
	Cxcl10	F : 5'-AAG TGC TGC CGT CAT TTT CT-3'<br>R : 5'-GTG GCA ATG ATC TCA ACA CG-3'
Housekeeping gene		
	Gapdh	F : 5'- TGG GCT ACA CTG AGC ACC AG -3'<br>R : 5'- GGG TGT CGC TGT TGA AGT CA -3'

SUPPLEMETARY MATERIALS AND METHODS FOR CHEMICAL SYNTHESIS

^{1}H and ^{13}C NMR spectra were recorded on a Varian Inova-500 (Varian Assoc., Palo Alto, USA), and chemical shifts were measured in ppm relative to internal tetramethylsilane (TMS) standard or specific solvent signal. Multiplicity was indicated as follows: s (singlet); d (doublet); t (triplet); q (quartet); m (multiplet); dd (doublet of doublet); dt (doublet of triplet); td (triplet of doublet); bs (broad singlet), etc. Coupling constants were reported in Hz. Low-resolution mass spectrometry (LRMS) was conducted by LC-MS-2020 (Shimadzu). High-resolution mass spectrometry (HRMS) of final compounds was confirmed by High-Resolution LC-MS/MS spectrometer Q-TOF 5600 (AB SCIEX) from the National Instrumentation Center for Environmental Management (NICEM) at Seoul National University.

All reagents in the synthetic procedure were purchased from Sigma-Aldrich (MO, USA) and TCI (Japan). The progress of the reaction was monitored using thin-layer chromatography (TLC) (silica gel 60 F_{254} 0.25 mm), and components were visualized by observation under UV light (254 and 365 nm) or by treating the TLC plates with anisaldehyde staining solution followed by heating. Silica gel 60 (40–63 μm) used in flash column chromatography was purchased from Merck (Germany). All reactions were conducted in oven-dried glassware under dry argon atmosphere unless otherwise specified. CH_2Cl_2 was distilled from CaH_2 immediately prior to use. Other solvents and organic reagents were purchased from commercial vendors and used without further purification unless otherwise mentioned.

SYNTHESIS AND CHARACTERIZATION OF COMPOUNDS

Scheme 1. General synthetic scheme of the compound **1–11**[a]

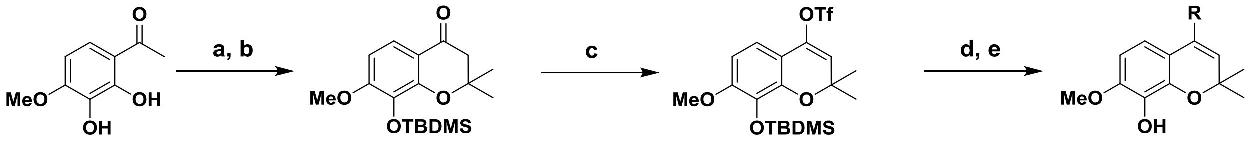

[a] Reagents and conditions: (a) acetone, pyrrolidine (2.0 equiv.), EtOH, reflux, overnight stirring; (b) TBDMSCl (1.2 equiv.), imidazole (1.5 equiv.), room temperature, 3 h; (c) trifluoromethanesulfonic anhydride (1.2 equiv.), 2,6-di-*tert*-butyl-4-methylpyridine (1.4 equiv.), CH_2Cl_2, 0 °C, 30 min; (d) $RB(OH)_2$ (1.1 equiv.), $Pd(PPh_3)_4$ (0.05 equiv.), Na_2CO_3 (2.0 equiv.), toluene / EtOH / H_2O, 70 °C, 3 h; (e) TBAF (1.1 equiv.), THF, room temperature, 30 min.

■ 4-(4-Chloro-2-methylphenyl)-7-methoxy-2,2-dimethyl-2H-chromen-8-ol (**1**)

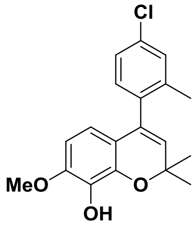

This compound was synthesized as previously reported.[1] LRMS (ESI^+) *m/z* calcd for $C_{19}H_{20}ClO_3$ $[M+H]^+$: 331.10; Found: 331.05.

■ 4-(4-Fluoro-2-methylphenyl)-7-methoxy-2,2-dimethyl-2H-chromen-8-ol (**2**)

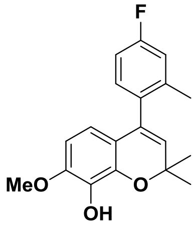

^1H NMR (500 MHz, $CDCl_3$) δ 7.09 (dd, *J* = 8 Hz, 6 Hz, 1H), 6.92 (m, 2H), 6.33 (d, *J* = 8.5 Hz, 1H), 6.08 (d, *J* = 8.5 Hz, 1H), 5.43 (s, 1H), 5.38 (s, 1H), 3.86 (t, *J* = 4.5 Hz, 3H), 2.15 (s, 3H), 1.55 (d, *J* = 14 Hz, 3H), 1.49 (d, *J* = 29.5 Hz, 3H); ^{13}C NMR (125 MHz, $CDCl_3$) δ 162.46, 148.00, 140.22, 139.31, 134.35, 133.99, 133.58, 131.39, 128.89, 127.88, 116.91, 116.79, 116.02, 112.71, 103.61, 56.32, 28.26, 27.89, 20.03; LRMS (ESI^+) *m/z* calcd for $C_{19}H_{20}FO_3$ $[M+H]^+$: 315.14; Found: 315.10.

■ 4-(4-Fluorophenyl)-7-methoxy-2,2-dimethyl-2H-chromen-8-ol (**3**)

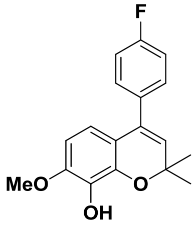

This compound was synthesized as previously reported.[1] LRMS (ESI^+) *m/z* calcd for $C_{18}H_{18}FO_3$ $[M+H]^+$: 301.12; Found: 301.10.

■ 7-Methoxy-2,2-dimethyl-4-(o-tolyl)-2H-chromen-8-ol (**4**)

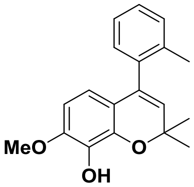

^{1}H NMR (500 MHz, $CDCl_3$) δ 7.19 (m, 3H), 7.13 (d, J = 1.5 Hz, 1H), 6.32 (d, J = 8.5 Hz, 1H), 6.11 (d, J = 8.5 Hz, 1H), 5.45 (s, 1H), 5.39 (d, J = 4.5 Hz, 1H), 3.84 (d, J = 2.5 Hz, 3H), 2.16 (s, 3H), 1.56 (s, 3H), 1.52 (s, 3H); ^{13}C NMR (125 MHz, $CDCl_3$) δ 147.93, 140.25, 138.16, 136.81, 134.42, 134.36, 130.18, 129.95, 127.91, 127.38, 125.95, 117.00, 116.20, 103.62, 56.32, 28.33, 27.94, 19.90; LRMS (ESI^+) *m/z* calcd for $C_{19}H_{21}O_3$ $[M+H]^+$: 297.15; Found: 297.10.

■ 4-(2,5-Dimethylphenyl)-7-methoxy-2,2-dimethyl-2H-chromen-8-ol (**5**)

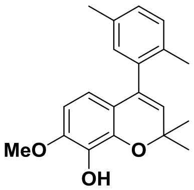

This compound was synthesized as previously reported.[1] LRMS (ESI^+) *m/z* calcd for $C_{20}H_{23}O_3$ $[M+H]^+$: 311.16; Found: 311.15

■ 7-Methoxy-4-(2-methoxyphenyl)-2,2-dimethyl-2H-chromen-8-ol (**6**)

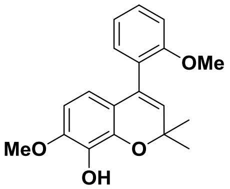

^{1}H NMR (500 MHz, $CDCl_3$) δ 7.33 (dd, J = 8 Hz, 2 Hz, 1H), 7.17 (dd, J = 8 Hz, 2 Hz, 1H), 6.96 (m, 2H), 6.33 (d, J = 8.5 Hz, 1H), 6.22 (d, J = 8.5 Hz, 1H), 5.48 (s, 1H), 5.42 (bs, 1H), 3.85 (d, J = 4.5 Hz, 1H), 3.72 (d, J = 5 Hz, 1H), 1.54 (s, 6H); ^{13}C NMR (125 MHz, $CDCl_3$) δ 157.65, 147.61, 140.16, 134.22, 132.00, 131.35, 129.34, 128.05, 127.73, 120.87, 116.98, 116.24, 111.46, 103.51, 56.31, 55.94, 28.11 (2C); LRMS (ESI^+) *m/z* calcd for $C_{19}H_{21}O_4$ $[M+H]^+$: 313.14; Found: 313.10.

■ 7-Methoxy-2,2-dimethyl-4-(quinolin-8-yl)-2H-chromen-8-ol (**7**)

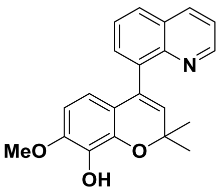

^{1}H NMR (500 MHz, $CDCl_3$) δ 8.87 (dd, J = 4 Hz, 2 Hz, 1H), 8.16 (dd, J = 8.5 Hz, 1.5 Hz, 1H), 7.83 (dd, J = 8 Hz, 1.5 Hz, 1H), 7.63 (dd, J = 7 Hz, 1.5 Hz, 1H), 7.56 (dd, J = 7.5 Hz, 1 Hz, 1H), 7.37 (dd, J = 8.5 Hz, 4 Hz), 6.22 (d, J = 9 Hz, 1H), 5.98 (d, J = 8.5 Hz, 1H), 5.63 (s, 1H), 5.48 (s, 1H), 3.81 (s, 3H), 1.62 (s, 6H); ^{13}C NMR (125 MHz, $CDCl_3$) δ 150.57, 147.61, 147.26, 140.07, 138.40, 136.24, 134.32, 133.55, 130.85, 128.58, 128.56, 128.36, 126.49, 121.35, 117.88, 116.79, 103.59, 56.31, 56.29, 28.33; LRMS (ESI^+) *m/z* calcd for $C_{21}H_{20}NO_3$ $[M+H]^+$: 334.14; Found: 334.10.

■ 4-(Dibenzo[b,d]furan-4-yl)-7-methoxy-2,2-dimethyl-2H-chromen-8-ol (**8**)

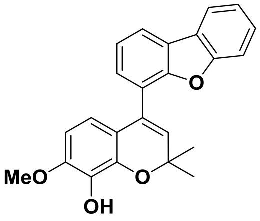

This compound was synthesized as previously reported.[1] HRMS (ESI$^+$) *m/z* calcd for $C_{24}H_{21}O_4$ [M+H]$^+$: 373.1434; Found: 373.1428.

■ 4-(Dibenzo[b,d]thiophen-4-yl)-7-methoxy-2,2-dimethyl-2H-chromen-8-ol (**9**)

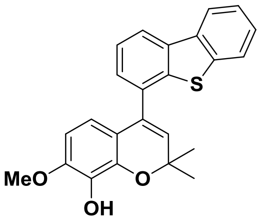

^{1}H NMR (500 MHz, $CDCl_3$) δ 8.16 (m, 2H), 7.79 (d, *J* = 7 Hz, 1H), 7.54 (m, 3H), 7.36 (d, *J* = 8.5 Hz, 1H), 6.33 (d, *J* = 4.5 Hz, 2H), 5.81 (s, 1H), 5.48 (s, 1H), 3.86 (d, *J* = 5 Hz, 1H), 1.62 (s, 6H); ^{13}C NMR (125 MHz, $CDCl_3$) δ 148.24, 140.672, 140.15, 139.82, 136.34, 135.98, 134.53, 133.48, 133.43, 128.59, 128.57, 127.48, 127.10, 124.82, 124.60, 122.93, 121.89, 121.07, 116.44, 115.71, 103.55, 56.32, 56.30, 27.96; LRMS (ESI$^+$) *m/z* calcd for $C_{24}H_{21}O_3S$ [M+H]$^+$: 389.12; Found: 389.05

■ 7-Methoxy-2,2-dimethyl-4-(thianthren-1-yl)-2H-chromen-8-ol (**10**)

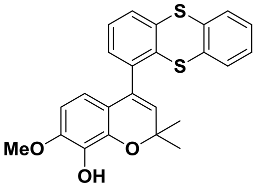

This compound was synthesized as previously reported.[1] LRMS (ESI$^+$) *m/z* calcd for $C_{24}H_{21}O_3S_2$ [M+H]$^+$: 421.09; Found: 421.05.

■ 4-([1,1'-Biphenyl]-3-yl)-7-methoxy-2,2-dimethyl-2H-chromen-8-ol (**11**)

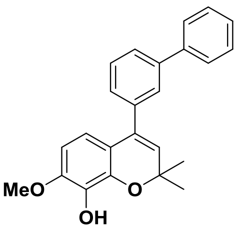

^{1}H NMR (500 MHz, $CDCl_3$) δ 7.60 (m , 4H), 7.44 (m, 3H), 7.34 (m, 2H), 6.58 (d, *J* = 8.5 Hz, 1H), 6.41 (d, *J* = 8.5 Hz, 1H), 5.59 (s, 1H), 5.47 (s, 1H), 3.88 (d, *J* = 4 Hz, 3H), 1.55 (d, *J* = 9.5 Hz, 6H); ^{13}C NMR (125 MHz, $CDCl_3$) δ 147.97, 141.54, 141.19, 140.78, 139.23, 135.02, 134.57, 129.03, 128.97, 127.90, 127.77, 127.65, 127.45, 127.44, 126.73, 116.84, 116.58, 103.68, 56.37, 56.34, 27.89; LRMS (ESI$^+$) *m/z* calcd for $C_{24}H_{23}O_3$ [M+H]$^+$: 359.16; Found: 359.10.

Scheme 2. General synthetic scheme for the compound **12** and **13**[a]

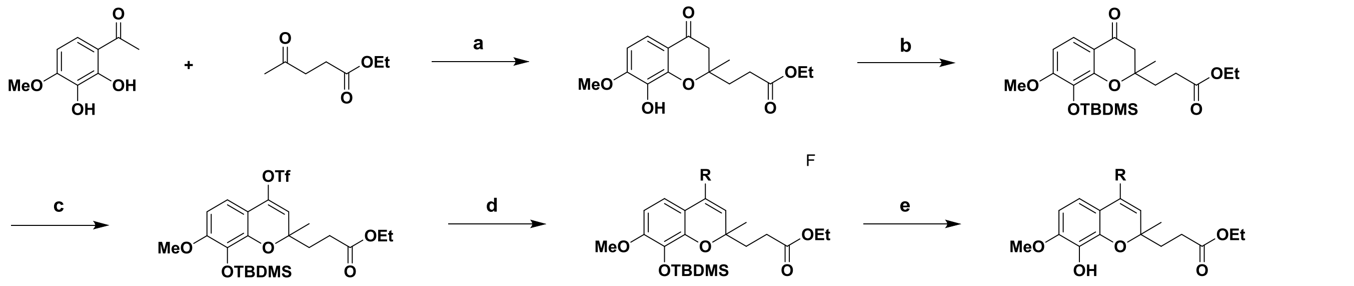

[a] Reagents and conditions: (a) pyrrolidine 2.0 (equiv.), EtOH, reflux, 3 days; (b) TBDMSCl (1.2 equiv.), imidazole (1.5 equiv.), room temperature, 5 h; (c) trifluoromethanesulfonic anhydride (1.0 equiv.), 2,6-di-*tert*-butyl-4-methylpyridine (1.4 equiv.), CH_2Cl_2, 0 °C, 30 min; (d) $RB(OH)_2$ (1.1 equiv.), $Pd(PPh_3)_4$ (0.05 equiv.), Na_2CO_3 (3.0 equiv.), toluene / EtOH / H_2O, 70 °C; (e) TBAF (1.1 equiv.), THF, 0 °C, 30 min.

- Ethyl 3-(4-(4-chloro-2-methylphenyl)-8-hydroxy-7-methoxy-2-methyl-2H-chromen-2-yl)propanoate (**12**)

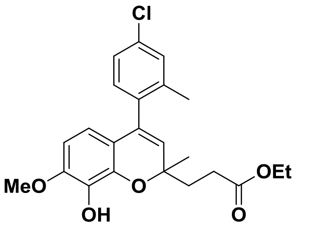

^{1}H NMR (500 MHz, $CDCl_3$) δ 7.23 (d, *J* = 3.5 Hz, 1H) 7.19 (t, *J* = 5.5 Hz, 1H), 7.06 (dd, *J* = 10.5 Hz, 8 Hz, 1H), 6.32 (d, *J* = 8.5 Hz, 1H), 6.07 (d, *J* = 8 Hz, 1H), 5.46 (s, 1H), 5.30 (d, *J* = 7 Hz, 1H), 4.10 (m, 2H), 3.86 (d, *J* = 5 Hz, 3H), 2.55 (m, 2H), 2.15 (m, 5H), 1.51 (d, *J* = 22 Hz, 3H), 1.24 (m, 3H); ^{13}C NMR (125 MHz, $CDCl_3$) δ 173.82, 148.30, 140.11, 139.94, 138.77, 138.71, 136.51, 136.39, 135.03, 134.70, 134.32, 134.16, 133.61, 133.55, 131.20, 131.12, 130.13, 130.08, 126.15, 126.11, 125.72, 125.70, 116.18, 116.07, 103.78, 103.69, 60.77, 56.33, 36.48, 36.16, 31.10, 29.97, 29.77, 26.75, 26.44, 19.98, 19.75, 14.41; LRMS (ESI$^+$) *m/z* calcd for $C_{23}H_{26}ClO_5$ $[M+H]^+$: 417.15; Found: 417.10.

- Ethyl 3-(4-(dibenzo[b,d]furan-4-yl)-8-hydroxy-7-methoxy-2-methyl-2H-chromen-2-yl)propanoate (**13**)

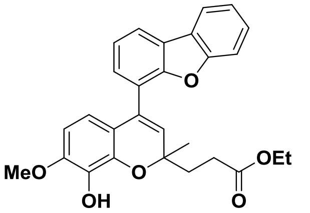

^{1}H NMR (500 MHz, $CDCl_3$) δ 7.96 (t, *J* = 8 Hz, 2H), 7.52 (d, *J* = 8.5 Hz, 1H), 7.44 (d, *J* = 7 Hz, 1H), 7.36 (m, 3H), 6.34 (dd, *J* = 22 Hz, 8.5 Hz, 2H), 5.71 (s, 1H), 5.54 (s, 1H), 4.11 (dd, *J* = 14 Hz, 7 Hz, 2H), 3.84 (s, 3H), 2.64 (m, 2H), 2.24 (m, 2H), 1.60 (s, 3H), 1.22 (t, *J* = 7.5 Hz); ^{13}C NMR (125 MHz, $CDCl_3$) δ 173.98, 156.34, 154.13, 148.25, 140.42, 134.33, 131.00, 128.29, 127.50, 127.45, 124.86, 124.41, 123.01, 122.96, 122.77, 120.87, 120.43, 116.63, 115.88, 112.20, 103.73, 60.75, 56.34, 36.32, 29.87, 26.51, 14.44; LRMS (ESI$^+$) *m/z* calcd for $C_{28}H_{27}O_6$ $[M+H]^+$: 459.18; Found: 459.15.

^{1}H and ^{13}C NMR Spectra

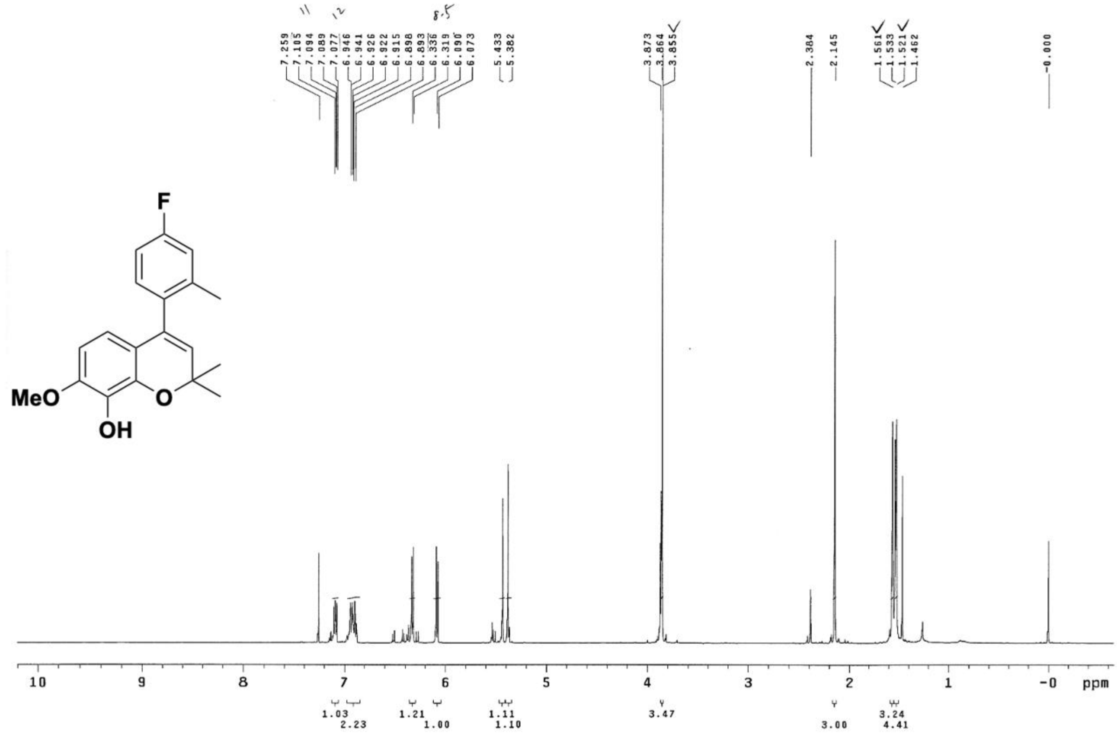

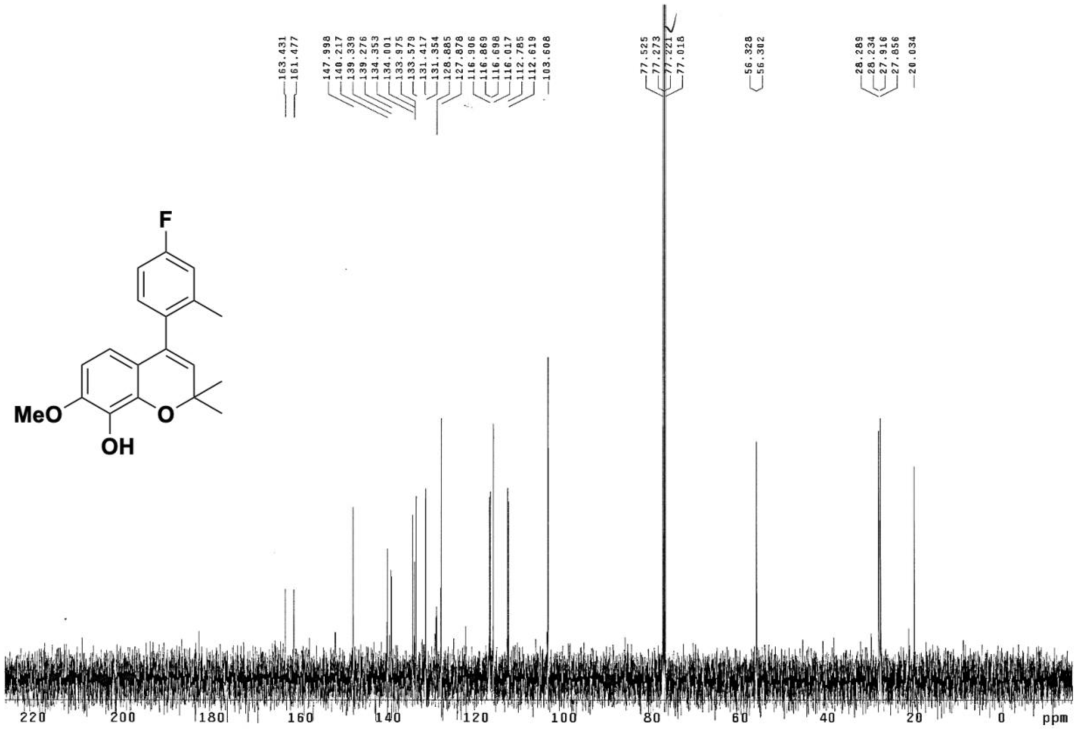

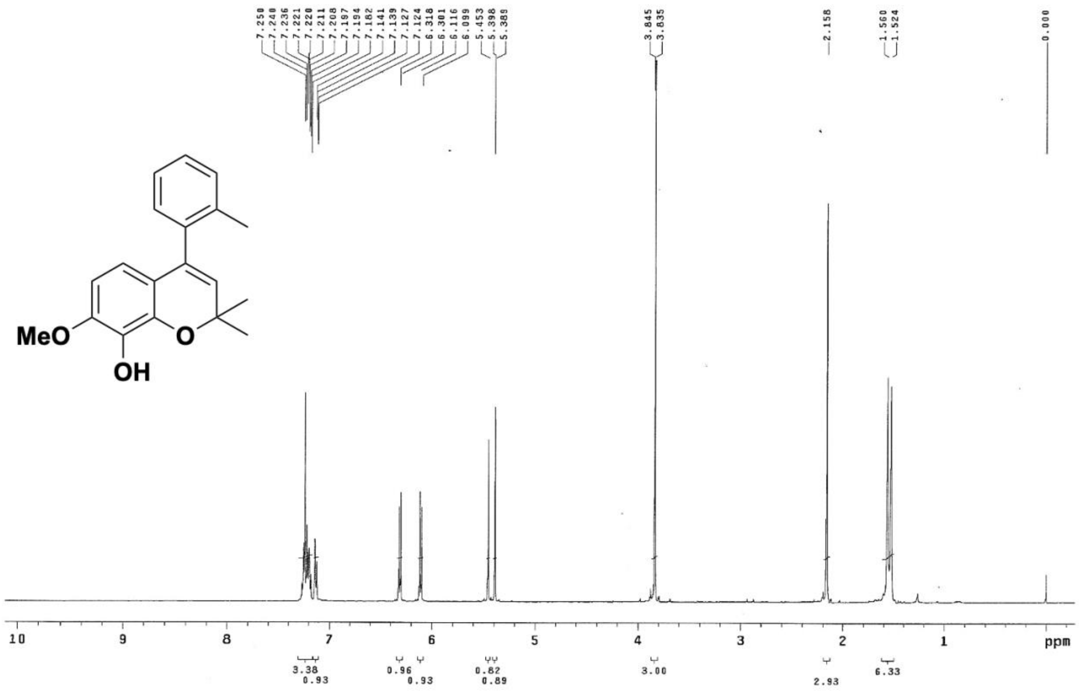

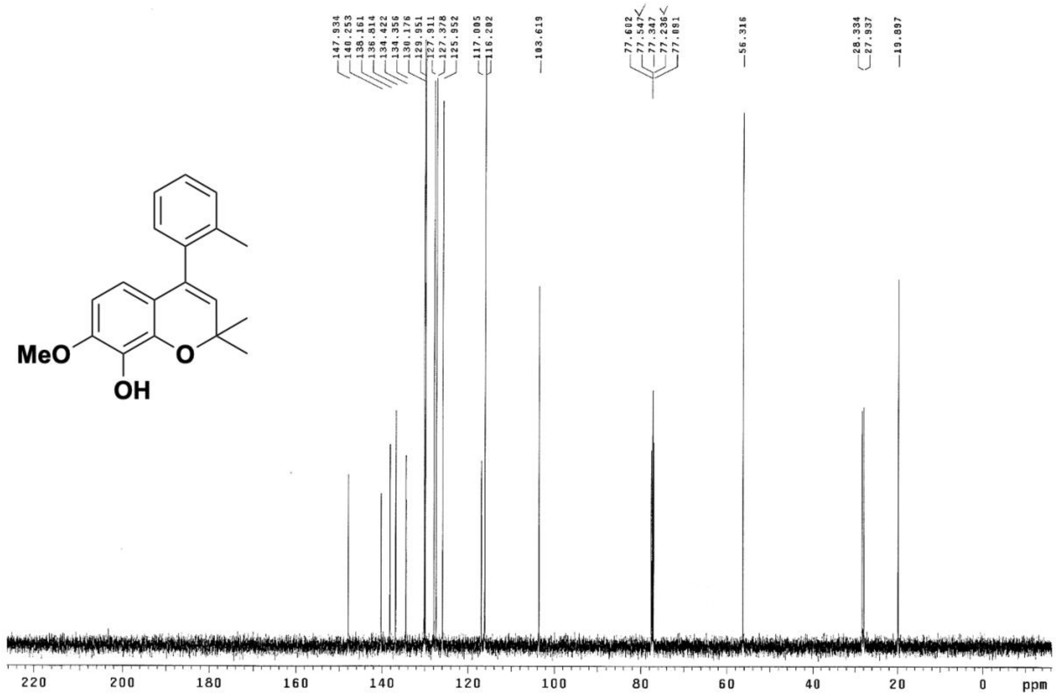

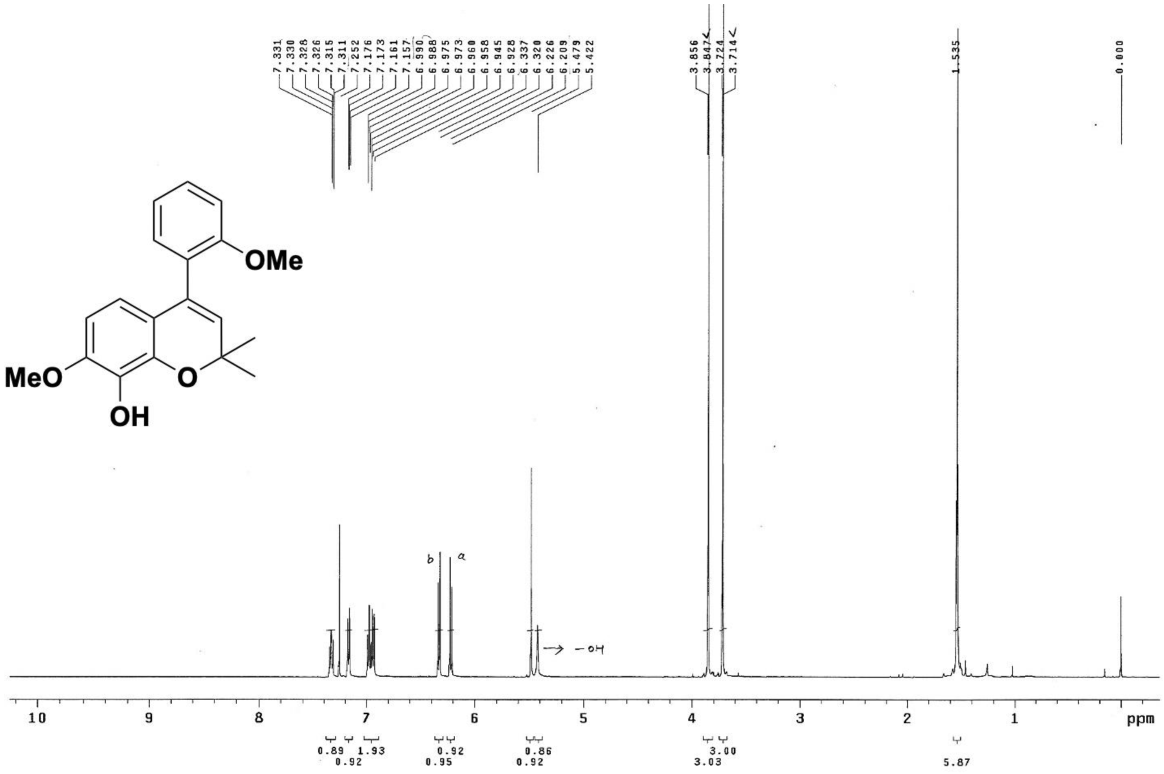
OMe
MeO
O
OH
ppm

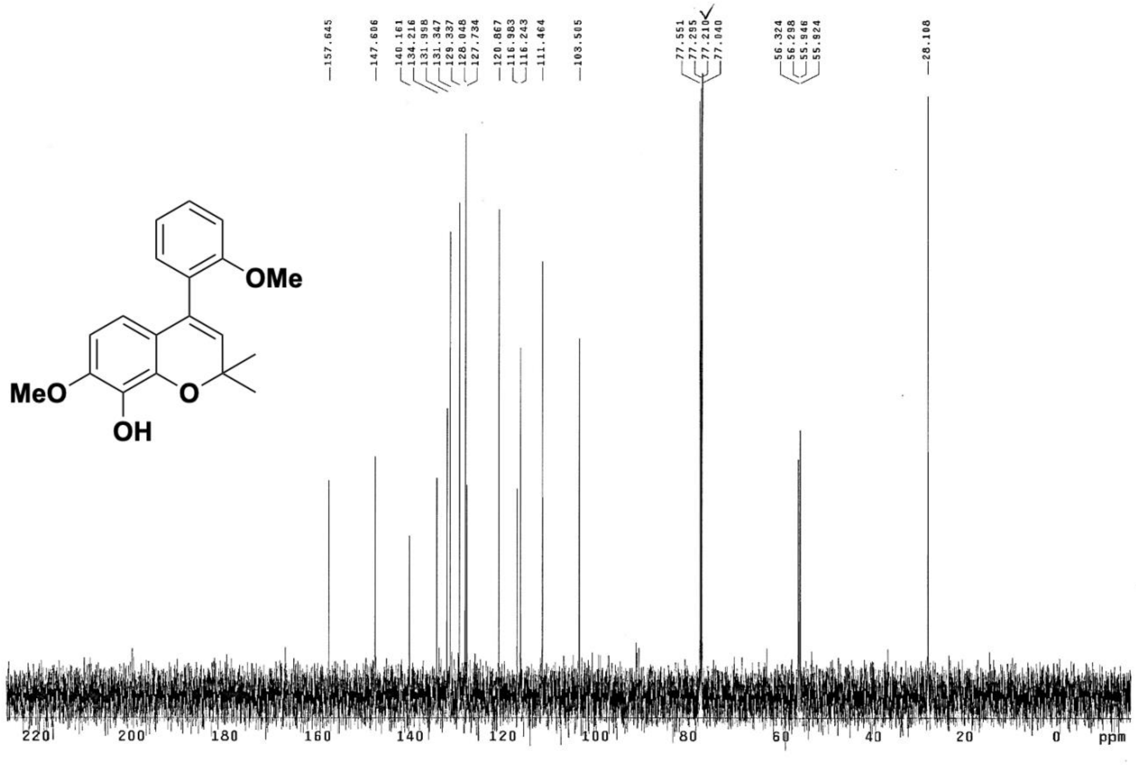
OMe
MeO
O
OH
ppm

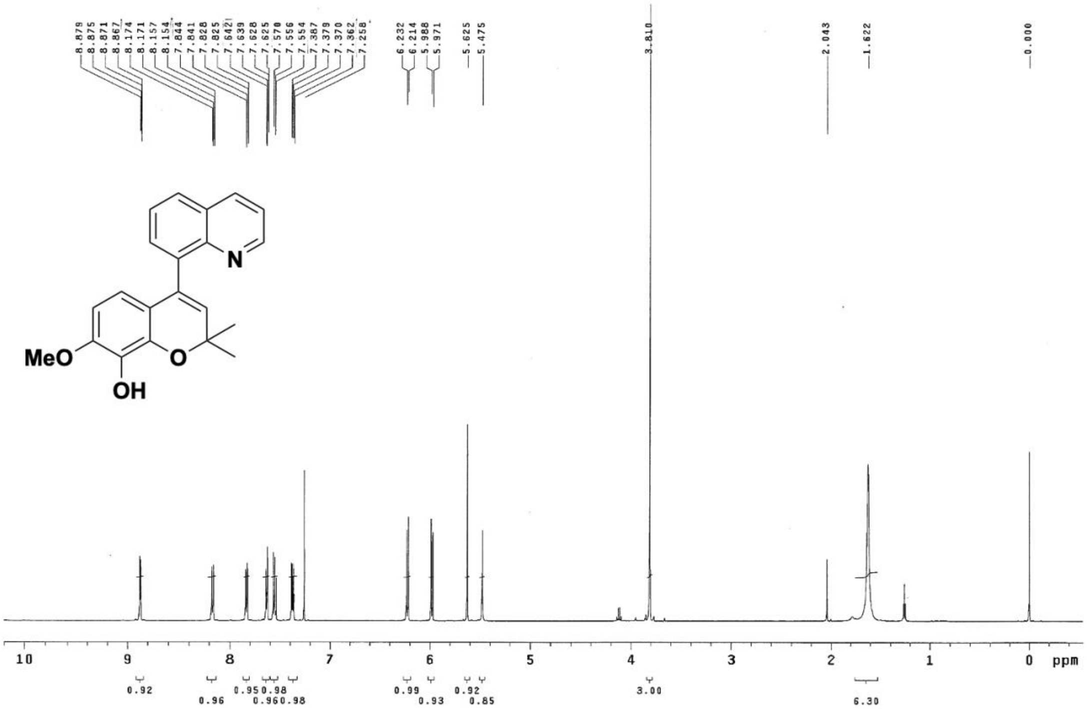
8.879
8.875
8.871
8.867
8.174
8.171
8.157
8.154
7.844
7.841
7.828
7.825
7.642
7.639
7.628
7.625
7.570
7.556
7.554
7.387
7.379
7.370
7.362
7.258
6.232
6.214
5.988
5.971
5.625
5.475
3.810
2.043
1.622
0.000
N
MeO
O
OH
10 9 8 7 6 5 4 3 2 1 0 ppm
0.92
0.96
0.95
0.98
0.96
0.98
0.99
0.93
0.92
0.85
3.00
6.30

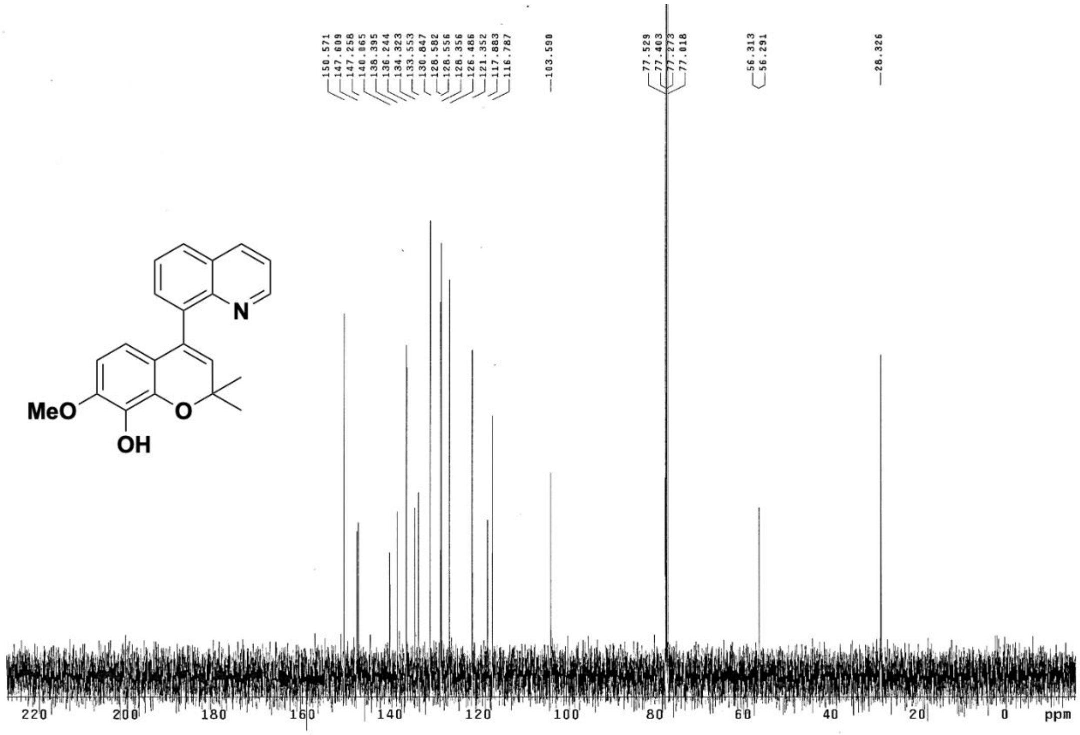
150.571
147.699
147.256
140.065
138.395
136.244
134.323
133.553
130.847
128.582
128.556
128.356
126.486
121.352
117.883
116.787
103.590
77.529
77.463
77.273
77.018
56.313
56.291
28.326
N
MeO
O
OH
220 200 180 160 140 120 100 80 60 40 20 0 ppm

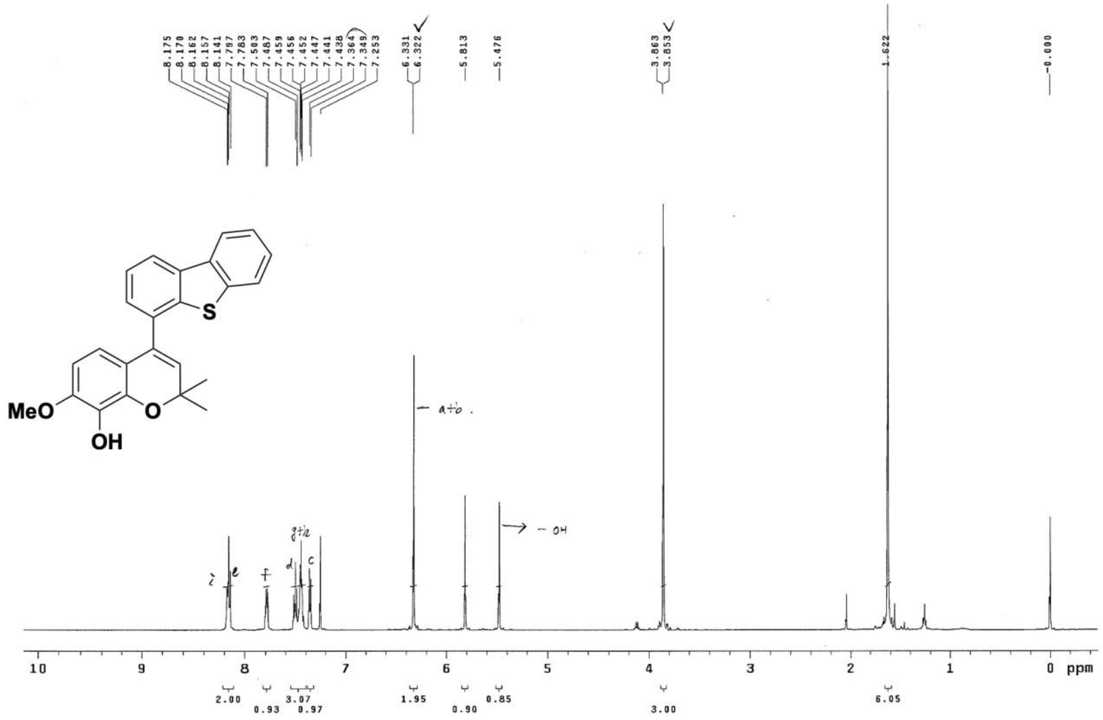
8.175
8.170
8.162
8.157
8.141
7.797
7.783
7.503
7.487
7.459
7.456
7.452
7.447
7.441
7.438
7.364
7.349
7.253
6.331
6.322
5.813
5.476
3.863
3.853
1.622
0.000
MeO
OH
O
S
2.00
0.93
3.07
0.97
1.95
0.90
0.85
3.00
6.05
ppm

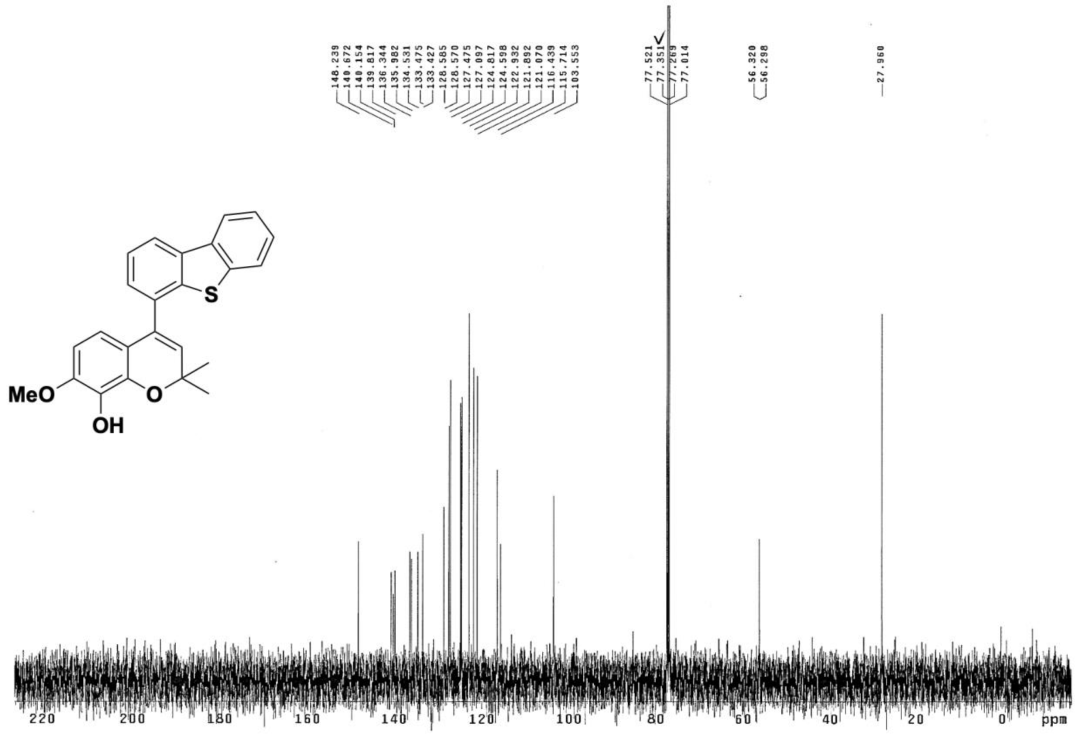
148.239
140.672
140.154
139.817
136.344
135.982
134.531
133.475
133.427
128.585
128.570
127.475
127.097
124.817
124.598
122.932
121.892
121.070
116.439
115.714
103.553
77.521
77.351
77.269
77.014
56.320
56.298
27.960
MeO
OH
O
S
ppm

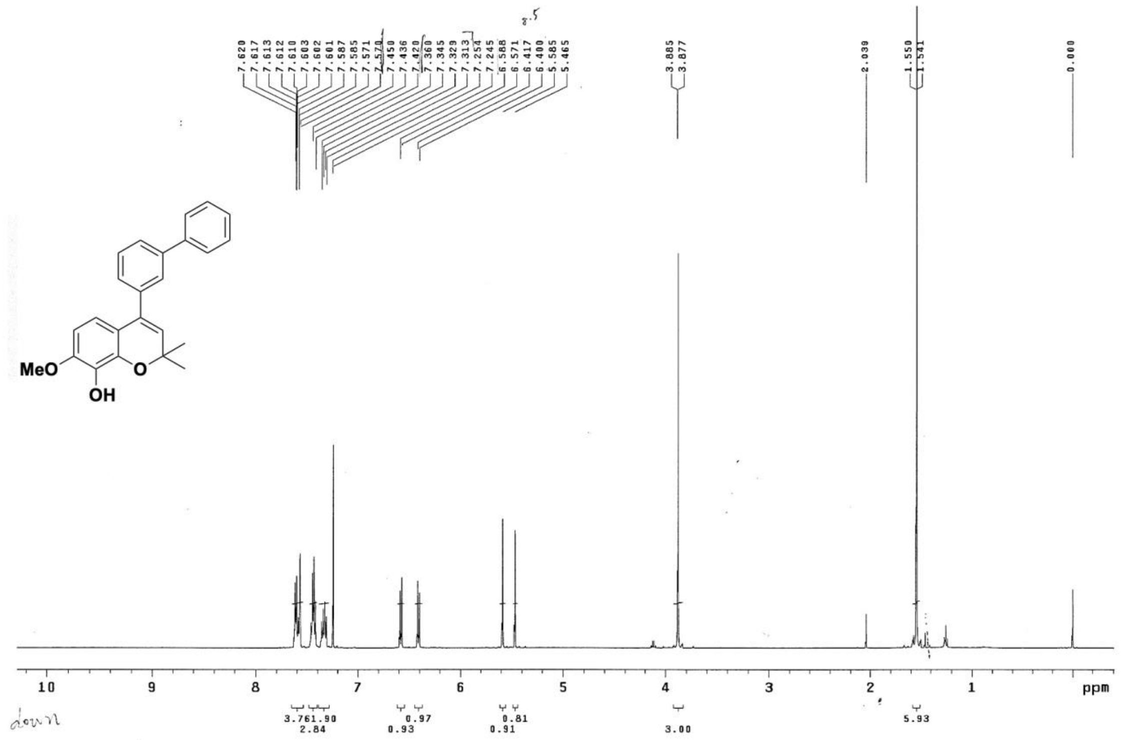
MeO
OH
O
ppm

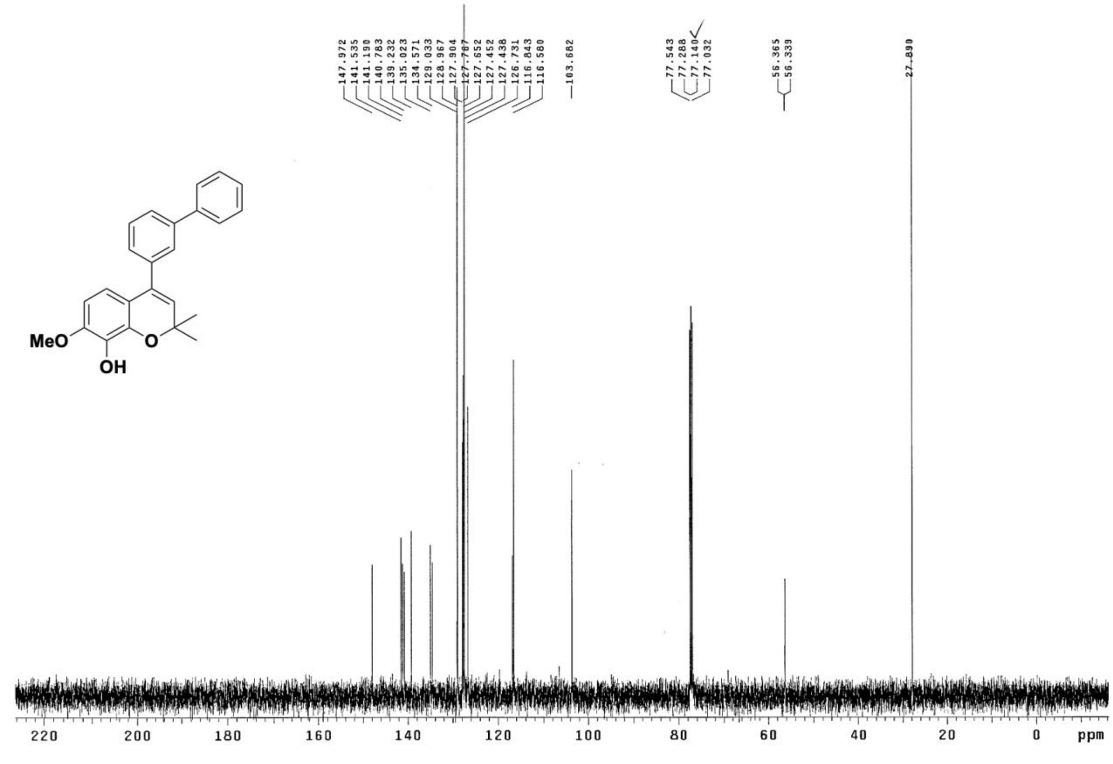
MeO
OH
O
ppm

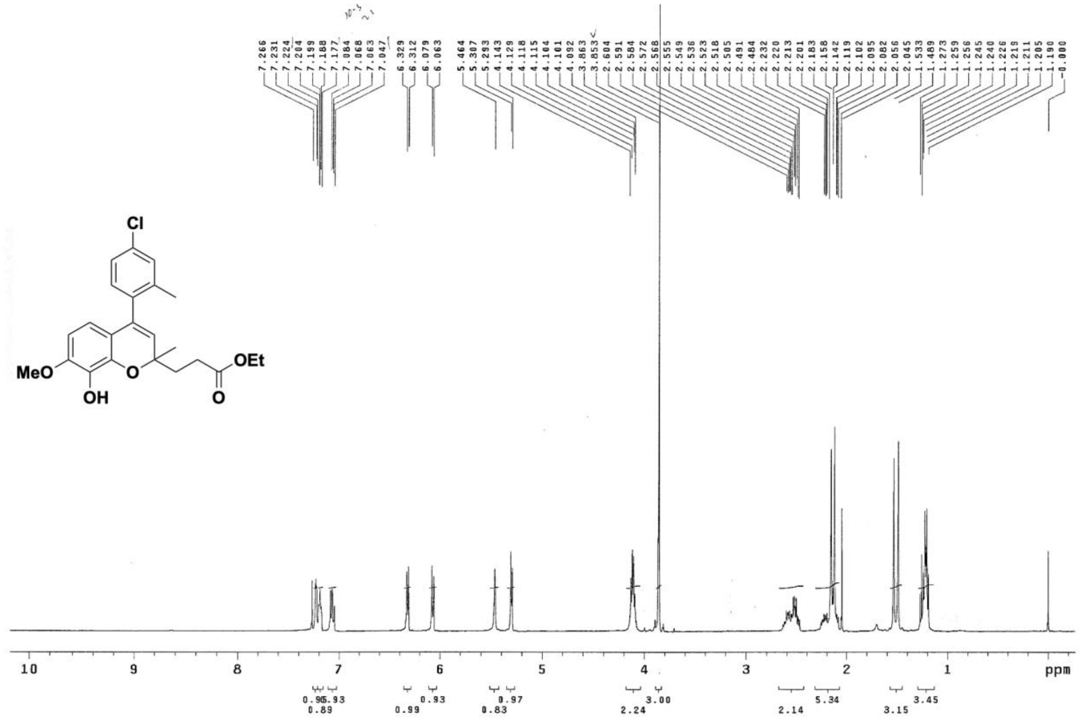
Cl
MeO
OH
O
OEt
O
ppm

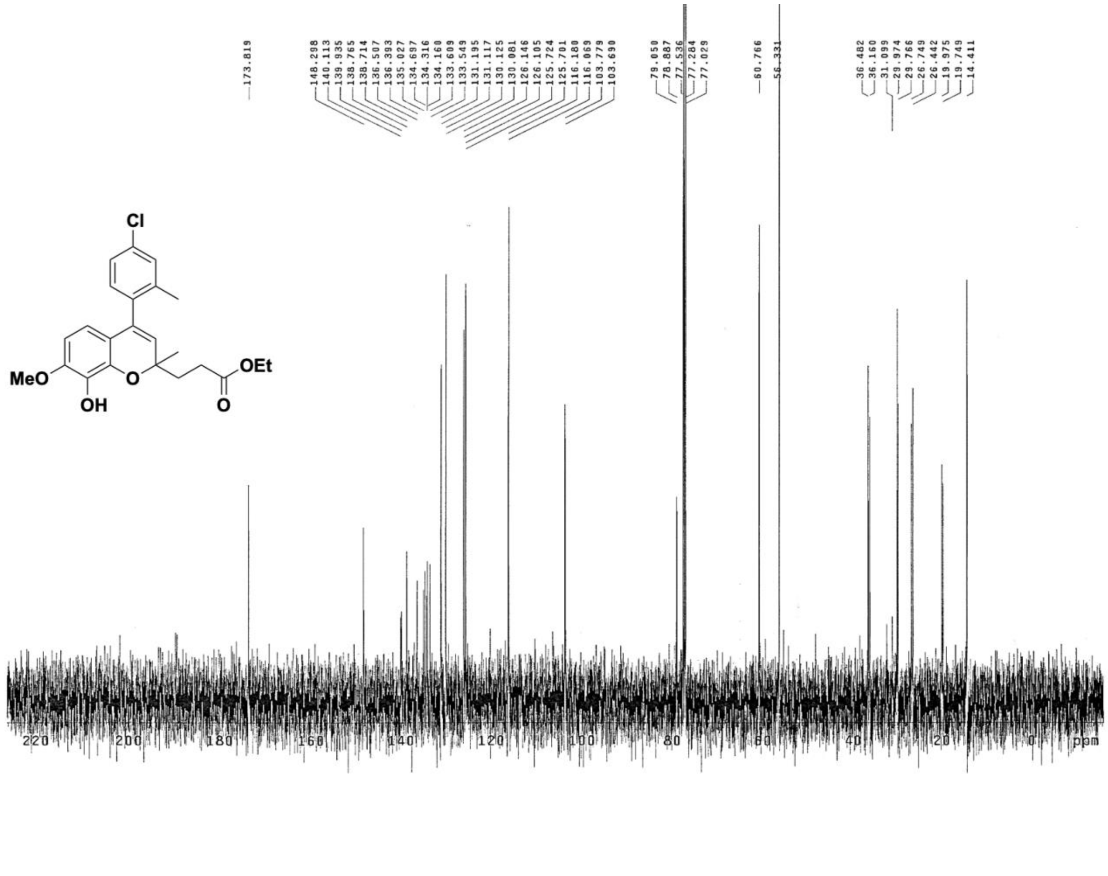
Cl
MeO
OH
O
OEt
O
ppm

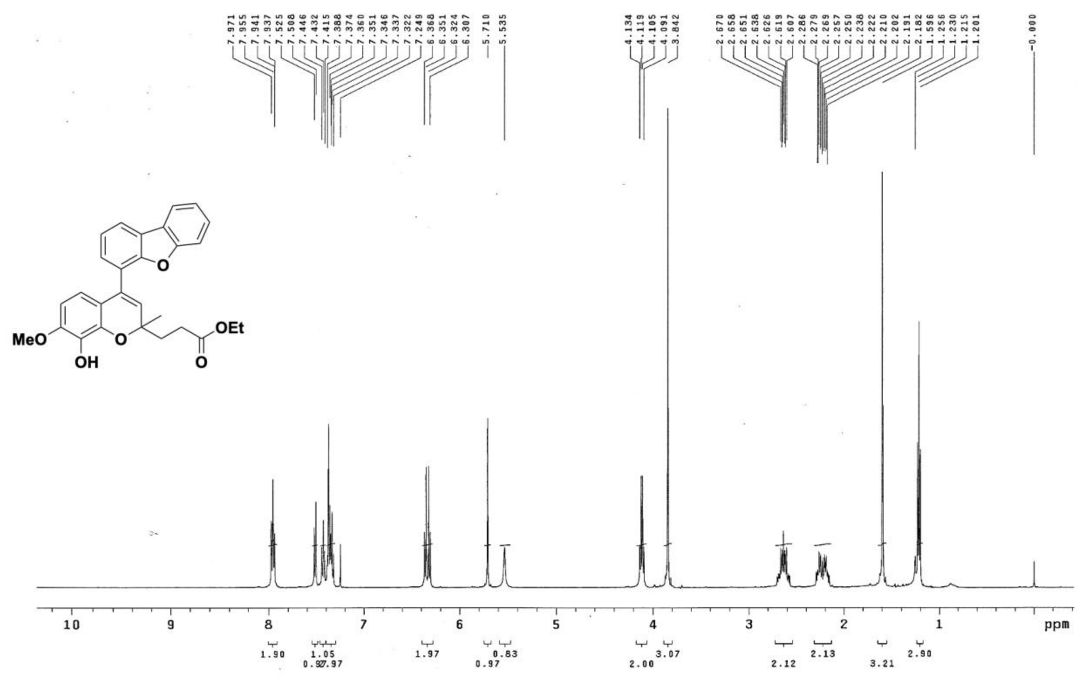

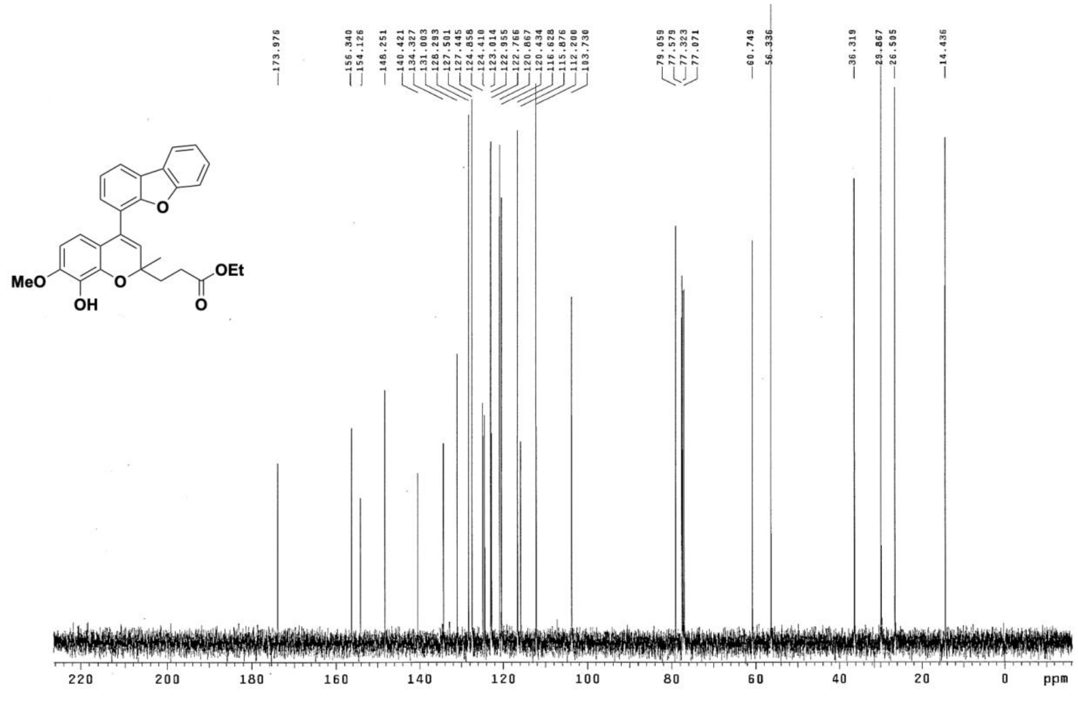

References

1. Oh, S., Jang, H. J., Ko, S. K., Ko, Y. & Park, S. B. *J. Comb. Chem* **12**, 548–558 (2010)
